# Surrogate-Assisted Optimization of Highly Constrained Oil Recovery Processes Using Classification-Based Constraint Modeling

**DOI:** 10.1021/acs.iecr.4c03294

**Published:** 2025-04-07

**Authors:** Zahir Aghayev, Dimitrios Voulanas, Eduardo Gildin, Burcu Beykal

**Affiliations:** † Department of Chemical and Biomolecular Engineering, University of Connecticut, Storrs, Connecticut 06269, United States; ‡ Center for Clean Energy Engineering, University of Connecticut, Storrs, Connecticut 06269, United States; § Harold Vance Department of Petroleum Engineering, Texas A&M University, College Station, Texas 77843, United States; ∥ Texas A&M Energy Institute, Texas A&M University, College Station, Texas 77843, United States

## Abstract

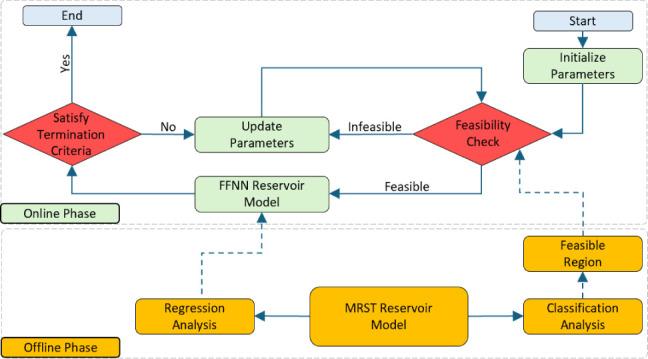

Real-world problems often involve constraints that must be carefully managed for feasible and efficient operations. In optimization, this becomes especially challenging with complex, high-dimensional problems that are computationally expensive and subject to hundreds or even thousands of constraints. We address these challenges by optimizing the highly constrained waterflooding process using a surrogate model of the reservoir and a classification-based constraint handling technique. Our study uses benchmark reservoir simulations, beginning with the low-dimensional Egg model and extending to the high-dimensional UNISIM model. We employ a Feedforward Neural Network (FFNN) surrogate for objective quantification and use classification-based modeling to transform the numerous constraints into a binary problem, distinguishing between feasible and infeasible reservoir settings. Our methodology involves an offline phase to develop and train models using reservoir simulation data, achieving high predictive accuracy (*R*^2^ > 0.98) with 20,000 bottom-hole pressure (BHP) settings and net present value (NPV) outputs. The classifier algorithms are then trained to model the constraints, ensuring that the solutions identified during optimization are feasible. In the online phase, we employ different model-based and search-based optimizers to find the optimal BHP settings that maximize the NPV throughout the production horizon. By integrating a highly accurate surrogate model and classification-based constraint handling, our approach significantly reduces the computational burden while ensuring that the solutions remain feasible, optimized for maximum economic gain, and yield better results compared to the deterministic approach.

## 1 Introduction

In 2023, global primary energy consumption hit a new record, increasing by 2%, marking a second consecutive year of record consumption. Despite significant growth in renewable energy capacity, fossil fuels still accounted for approximately 81.5% of the total energy mix.^1^ This continued reliance on fossil fuels underscores the ongoing importance of crude oil, a crucial component of the global energy supply. Innovations in oil extraction and production technologies are essential for maximizing efficiency and prolonging the life of existing oil fields. As new oil discoveries become rarer, improving recovery techniques from existing reserves is increasingly critical. These methods sustain production levels and contribute to energy security and economic stability by using existing resources better and reducing the environmental impact.

Oil recovery is categorized into primary, secondary, and tertiary stages.^2^ Primary recovery, driven by natural reservoir pressure, typically yields less than 30% of the original oil in place (OOIP). Relying solely on primary recovery is inadequate, necessitating secondary recovery methods like waterflooding to recover an additional 10–20% of OOIP. Enhanced oil recovery (EOR) employs advanced techniques to extract an extra 20–30% of OOIP.^3^ Secondary recovery supplements the reservoir’s energy by water or gas injection, driving additional crude oil out using pressure maintenance techniques such as waterflooding and gas injection.^4,5^ In waterflooding, water is injected into a reservoir using injection wells to maintain pressure, creating a gradient that displaces the oil toward production wells. While EOR methods can significantly increase oil recovery, they are costly and not always cost-effective, particularly at low oil prices.^6,7^ Waterflooding stands out due to its simplicity, cost-effectiveness, and ability to maintain reservoir pressure, making it a highly attractive oil recovery technology.

Despite its critical role in maximizing oil production, managing water during waterflooding is complex and challenging. Globally, the produced water (PW) volume reached approximately 250 million barrels per day in 2020, with PW exceeding oil production at a volumetric ratio of about 2.4.^8^ In the United States alone, nearly 24.4 billion barrels of PW are produced annually from around 1 million oil and gas wells, highlighting the scale of the issue.^9−11^ This enormous volume of PW includes formation water, injection water, and minor amounts of condensed water from gas production, with 40% of this PW being discharged untreated into the environment.^12^ The global produced water market is predicted to reach $87.4 billion by 2031.^13^ The high costs associated with managing this produced water underscore the need for more efficient and sustainable management practices. Optimizing the waterflooding process is crucial not only for effective water management and minimizing water usage but also for addressing significant economic and environmental concerns, ensuring a more sustainable approach to meeting global energy demands.

Numerous studies have explored optimizing waterflooding operations using mathematical modeling and computational reservoir simulations to determine the optimal well pressures or flow rates for maximizing cumulative oil production. Researchers have employed both gradient-based methods and data-driven optimization strategies to address the waterflooding control problem.^14−16^ Gradient-based approaches are computationally efficient but often get trapped in local minima.^17,18^ In contrast, data-driven optimization methods have gained popularity for their ability to explore complex search spaces without relying on derivative information on the original model, which can help mitigate the risk of getting trapped in local optima. These methods, including heuristic algorithms like differential evolution (DE),^19^ particle swarm optimization (PSO),^20^ and covariance matrix adaptation evolution strategy (CMA-ES),^21^ have shown superior performance in complex optimization tasks, making them highly suitable for well control optimization in waterflooding operations.

Harding et al.^22^ were among the first to document the use of data-driven optimization for well control schedules to maximize the Net Present Value (NPV) of reservoirs. They reviewed several optimization strategies, including stochastic search, simulated annealing, genetic algorithms (GA), and sequential quadratic programming (SQP). This initiated a wave of studies that tackled the waterflooding problem with bound constraints on decision variables, developing algorithms to manage the computational complexities involved. For instance, Cullick et al.^23^ applied a metaheuristic scatter search algorithm for production scheduling in synthetic oil fields. Wang et al.^24^ used a Kalman filter approach to optimize bound-constrained waterflooding, accounting for geological uncertainties. Zhao et al.^25^ utilized the NEWUOA algorithm and quadratic interpolation to maximize NPV within bound constraints on control variables.

Asadollahi et al.^26^ compared five black-box optimization techniques, including generalized pattern search (GPS), Hooke–Jeeves, Nelder–Mead, SQP, and line-search derivative-free algorithms, for optimizing production in the Brugge field under bound constraints. Golzari et al.^27^ employed artificial neural networks (ANNs) to develop a surrogate reservoir model, later used to maximize NPV in waterflooding. Azamipour et al.^28^ optimized water injection rates using a hybrid GA-polytope method. Wang et al.^29^ used GPS, PSO, and CMA-ES in a multiscale framework for oil well control optimization. Chen et al.^30^ developed the SADE-Sammon method, which integrates surrogate-assisted evolutionary algorithms with Sammon mapping for dimensionality reduction, showing superior performance on two numerical reservoir models. Kim and Durlofsky^31^ developed a recurrent neural network-based surrogate model for waterflooding optimization. Ng et al.^32^ used ANNs to create proxy models for optimizing well control rates, employing PSO and GWO on 2D and 3D reservoir models. Reginato et al.^33^ applied a hybrid machine learning (HML) approach using K-means and ANNs to predict petrophysical behaviors and optimize NPV during engineered water injection (EWI), significantly enhancing oil production and reducing water use.

Recent studies have also explored the use of reinforcement learning (RL) in waterflooding optimization. Miftakhov et al.^34^ used deep RL to optimize water injection rates based on grid pressure and water saturation data. Ma et al.^35^ applied deep RL for waterflooding optimization under geological uncertainties. He et al.^36^ used the proximal policy optimization algorithm to find optimal drilling schemes for greenfield primary depletion. Zhang et al.^37^ created an effective deep reinforcement learning (DRL) agent for life-cycle waterflooding production optimization. Hourfar et al.^38^ demonstrated that RL could effectively adjust water injection rates for different production policies and timelines, outperforming gradient-based and reactive control strategies regarding economic gain.

Despite advancements in optimization techniques, many do not adequately handle the general constraints critical for successful waterflooding management. Real-world problems often involve hundreds or even thousands of constraints, all of which must be managed to ensure a viable process. Echeverría Ciaurri et al.^39^ conducted a comparative study of various black-box optimization solvers and constraint-handling techniques, such as penalty functions, filter methods, and parameterless penalty functions. Other studies have explored various constraint-handling methods, including filter-based approaches,^40,41^ penalty functions,^42^ augmented Lagrangian techniques,^43^ and feasibility rule-based strategies.^44^ Constraints are not just a procedural necessity but a fundamental aspect of optimization that ensures solutions are both optimal and feasible within real-world conditions. These constraints typically involve physical, economic, and operational factors that are essential for practical outcomes. In waterflooding optimization, neglecting water-related constraints can lead to overestimated oil production and economic benefits. Our previous work showed that ignoring these constraints inflated the objective function (NPV), emphasizing the need for their inclusion to produce realistic and actionable results.^45^

To guarantee feasibility in waterflooding optimization, recent studies have focused on explicitly modeling constraints. We have previously used linear and nonlinear surrogate functions to approximate the objective function and constraints using a regression approach for flow rate, platform capacity, and water-cut constraints for the UNISIM case study.^45^ However, this traditional explicit modeling approach, while guaranteeing feasibility, does not scale well with the increasing complexity and dimensionality of reservoir simulations. In response to these challenges, classification-based feasibility modeling has emerged as a promising strategy. Classification algorithms have shown promising results in various domains for different applications by classifying the input space into different classes to predict outcomes, identify patterns, and ensure feasibility.^46−52^ By transforming the input space into binary feasible and infeasible regions accurately, this approach can theoretically map the feasible region of any number of constraints in one-go, while also handling the computationally expensive objective function evaluation by building accurate surrogate models.^53−58^ Yet, the applicability of this technique has only been shown in relatively small dimensional problems (10 variables and 5 constraints in ref (53)) or for constructing a single feasibility constraint based on simulation convergence, as demonstrated in ref (58), whereas the performance and the classification accuracy of high dimensional and highly computationally expensive problems still need to be quantified.

Motivated by this, we propose a computational framework that optimizes high-dimensional reservoir simulations by using a regression model to replicate key simulation outputs while implicitly managing thousands of operational constraints through classification algorithms. Our hybrid regression-classification approach aims to deliver superior objective values by simultaneously modeling and filtering out infeasible solutions, thereby enabling consistent convergence to feasible reservoir settings with significant computational efficiency gains. Unlike our previous investigation, which relied solely on regression with polynomials and interpolating functions, this study integrates deep learning and other advanced machine learning models to effectively map the feasible region of reservoir operations.^45^ We demonstrate that this integrated approach yields higher economic gains, better computational efficiency, and a feasible operation schedule for the waterflooding process compared to traditional deterministic optimization approaches. We apply this approach to a synthetic reservoir simulation, involving 312 control variables and 416 output constraints, and then extend it to a more complex real-world reservoir with 1,525 control variables and 1,769 output constraints. The process is first optimized deterministically using the methodology from the previous study, which utilized global optimization of a grey-box system with explicit constraint handling. The deterministic results are then compared with those from the current data-driven optimization approach.

## 2 Methodology

### 2.1 Case Studies

The proposed methodologies in this study are applied to optimize waterflooding processes using reservoir simulations. These simulations are based on fundamental principles of fluid flow in porous media, primarily governed by Darcy’s law, which relates flow rate to pressure gradient, fluid viscosity, and rock permeability. In reservoir conditions, multiple fluid phases (oil, gas, and water) coexist, requiring complex multiphase flow models incorporating concepts such as relative permeability and capillary pressure.^59−61^ To assess our approach’s effectiveness, we examine two benchmark reservoir models: the low-dimensional Egg model and the high-dimensional UNISIM model. These case studies allow the evaluation of our optimization techniques across different scales and complexities of reservoir simulations.

#### 2.1.1 Egg Model

**Figure 1:**
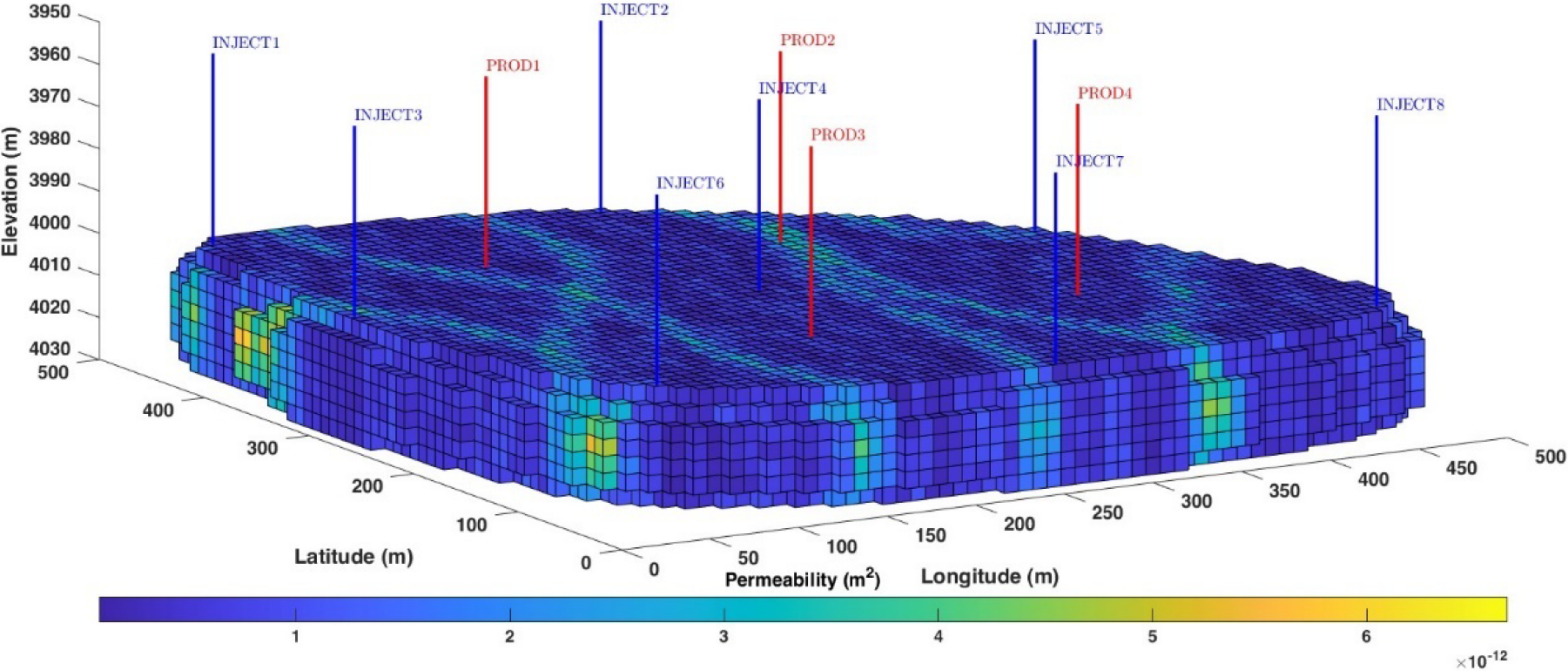
Permeability distribution of the Egg reservoir simulation. Injector wells are in blue, and producer wells are in red.

The Egg model (Figure 1) is a synthetic reservoir model with an ensemble of synthetic permeability fields. It includes 101 discrete permeability realizations of a channelized oil reservoir with eight water injectors and four oil producers. It has been used to test algorithms for computer-assisted flooding optimization, history matching, or, in combination, closed-loop reservoir management. This model comes with 25,200 grid cells. The active 18,553 cells form an egg-shaped model. High-permeability channels within a low-permeability background exemplify typical meandering river patterns found in fluvial environments. The fields exhibit a clear channel orientation, characterized by typical channel spacing and sinuosity. The permeability values are not conditioned on the wells, while porosity is assumed to be constant. Readers are referred to ref (62) for more information about the Egg model.

#### 2.1.2 UNISIM Model

**Figure 2:**
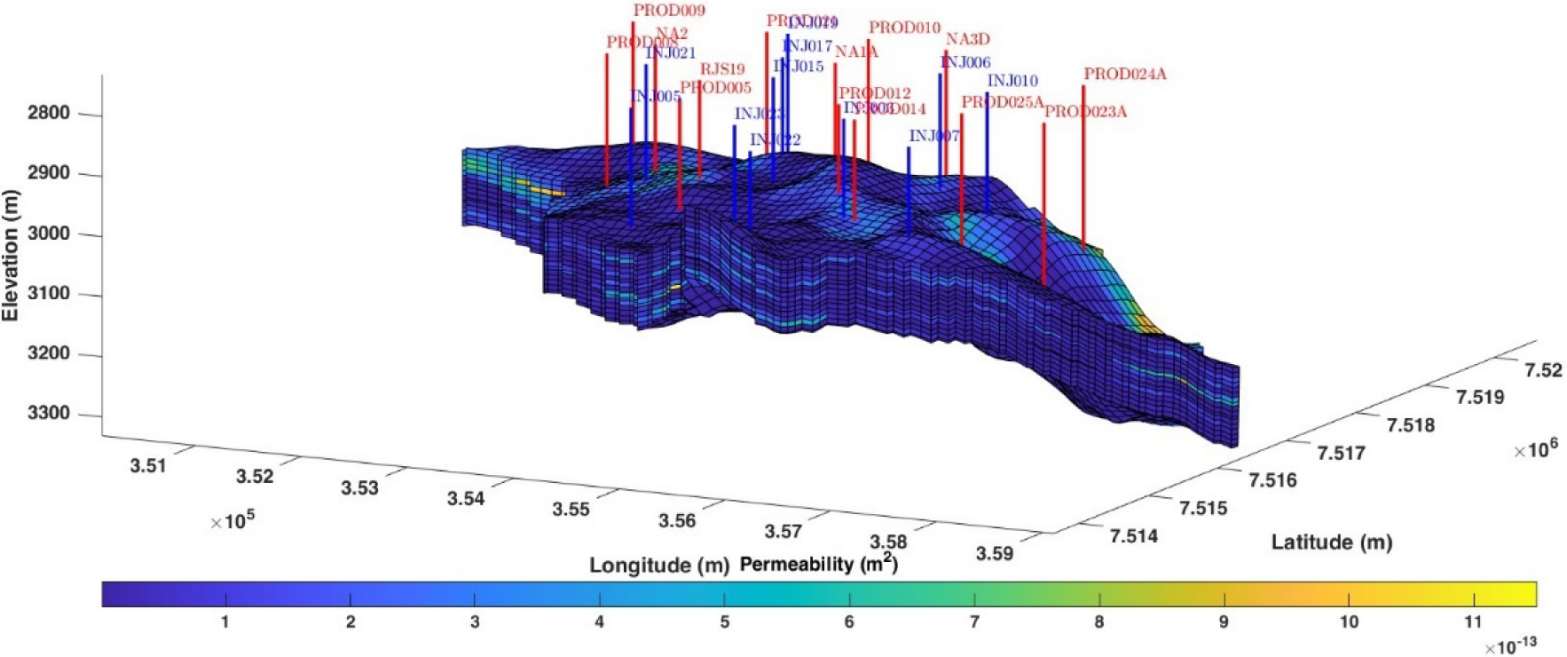
Permeability distribution of the UNISIM reservoir simulation. Injector wells are in blue, and producer wells are in red.

The UNISIM model (Figure 2) is constructed based on structural, facies, and petrophysical data from the Namorado field, Brazil. The structural elements include top, bottom, reservoir limits, and faults. The construction of this synthetic reservoir model aimed to create a high-resolution grid that can be used in numerical simulation and management-integrated studies of petroleum reservoirs. There are 25 wells (4 vertical producers, 10 horizontal producers, and 11 injectors). Thus, enabling testing and comparison of methodologies applied by different research groups. A grid 25 × 25 × 1 m cell resolution was discretized into a corner point grid with 326 × 234 × 157 cells with 3,408,633 active total cells to predict reservoir performance while considering small-scale heterogeneities. Readers are referred to ref (63) for more information about the UNISIM model.

### 2.2 Waterflooding Optimization Problem

The production optimization problem in waterflooding aims to maximize financial profit by optimizing the control scheme of each well. This involves adjusting either the bottom hole pressure (BHP) or the flow rate of each well. In our approach, we focus on the BHP control scheme as it allows direct management of reservoir pressure distribution and simplifies the handling of constraints, reducing problem complexity. Additionally, BHP-based controls are widely used in field operations due to their compatibility with existing equipment, such as downhole sensors, making them practical and efficient to implement.^59,64,65^ The objective is to maximize the Net Present Value (NPV) of the waterflooding project by periodically adjusting the BHP schedule of each well over the production time interval. This can be formulated as
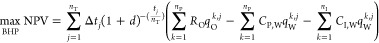
1where *n*_T_ is the total number of time steps, Δ*t*_*j*_ is the time interval at each time step, *d* is the discount rate, *R*_0_ is the revenue from oil production, *C*_P,W_ is the cost associated with produced water, *C*_I,W_ is the cost of injected water, *q*_0_ is the volumetric flow rate of oil, and *q*_W_ is the volumetric flow rate of water. The subscripts *i* and *p* are used to denote injectors and producers, respectively, ensuring that each term in the summation is aligned with the corresponding well type.

The optimization problem focuses on determining the optimal control variables over the entire production horizon. At each time step, these control variables are the BHPs for each well (injectors and producers). Specifically, the number of control variables is *d* = (*n*_I_ + *n*_P_) × *n*_T_, where *n*_I_ is the number of injectors and *n*_P_ is the number of producers. This means the total number of decision variables is the product of the number of wells and time steps (*n*_T_).

The dimensionality of the discretized optimal control problem depends on the time step size, total production time horizon, and the number of wells. As the time step becomes smaller or the production horizon extends, the number of optimization variables grows significantly. For example, the Egg model includes 8 injectors and 4 producers, while the UNISIM model features 11 injectors and 14 producers. The waterflooding optimization for the UNISIM model is conducted over a 5-year horizon with monthly control adjustments, resulting in 61 measurements. In contrast, the Egg model is optimized over a 0.5-year horizon with weekly adjustments, leading to 26 measurements. Additionally, controlling multiple injectors and producers simultaneously in realistic scenarios further increases the complexity and dimensionality of the search space.

To escape from the “curse of dimensionality", we use the functional control method (FCM) to parametrize the problem by representing the well-controlled domain with surrogate functions.^45,66^ Essentially, the BHP trajectory of each well is modeled using a continuous function of time. FCM allows for any suitable functional form to approximate the control trajectory. In this study, we use a second-order polynomial function for its simplicity and effectiveness in capturing the pressure control profiles observed in waterflooding applications (eqs 2 and 3). The second-order polynomial not only reduces the dimensionality but also ensures that the optimal pressure control profiles are smooth, avoiding abrupt changes between time steps that could lead to operational issues.
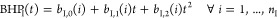
2
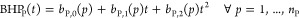
3

By defining the surrogate function with a manageable number of parameters, we significantly reduce the number of decision variables. The polynomial parameters concisely represent the control trajectory, thus simplifying the optimization process. For instance, the zeroth-order parameter indicates the initial pressure level, while the first and second-order parameters represent the trajectory’s rate of change and curvature, respectively. This method also allows us to set bounds on these parameters to control the rate of change in the pressure levels, ensuring they stay within practical limits. Specifically, eqs 4–6 define the lower and upper bounds for these parameters
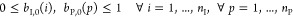
4
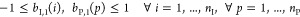
5
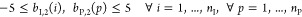
6

The main advantage of this approach is that it transforms the high-dimensional optimization problem into a more computationally feasible one. Instead of optimizing individual pressure levels at each time step, we optimize the surrogate function BHP(*t*) parameters. This reduction in complexity is significant: for the UNISIM model, the original optimization problem involves 1,525 pressure control variables (61 *measurements* × 25 *wells*) which is reduced to just 75 parameters. Similarly, the (26 *measurements* × 12 *wells*) to only 36 parameters. This simplification enables more efficient and effective optimization of waterflooding operations.

The optimization formulation for waterflooding involves several key constraints to ensure feasibility and operational efficiency. The total number of constraints is influenced by the control frequency and the length of the time horizon; more frequent control and extended periods significantly increase the number of constraints.

First, there are pressure constraints on the BHP for both injectors and producers to ensure safe and efficient operations. These constraints are given by
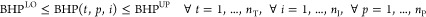
7

Flow rate constraints limit the amount of water and total liquid (water and oil) that each injector and producer can process to maintain operational feasibility
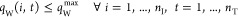
8
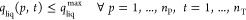
9where *q*_liq_ is the volumetric flow rate of liquid.

Platform capacity constraints ensure that the total production and injection rates do not exceed platform limits for water and oil
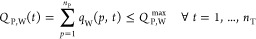
10
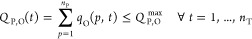
11
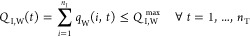
12where *Q*_P,W_ is the platform capacity for water in producers, *Q*_P,O_ is the platform capacity for oil in producers, and *Q*_I,W_ is the platform capacity for water in injectors.

The water-cut constraint (WC) limits the amount of water produced relative to the total liquid production (oil + water). This assists in maintaining the project’s economic viability since excessive water production increases operational costs
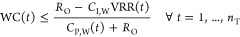
13where WC(*t*) is the water-cut at time *t*, defined as the fraction of water in the total liquid production. The term VRR(*t*), known as the voidage replacement ratio at time *t*, represents the ratio of the volume of the injected fluid (water) to the total volume of produced fluid (oil + water) at time *t*. It is calculated as
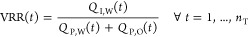
14

A VRR(*t*) close to 1 indicates balanced operations, where the reservoir pressure is stabilized by compensating for the produced fluid volumes with injected water. Values significantly above or below 1 can indicate operational inefficiencies such as water leakage outside the reservoir or reservoir damage (e.g., the fracturing formation).

These constraints are essential for ensuring the optimization process adheres to operational and economic limits and maintaining the solution’s feasibility. The number of constraints is directly determined by the number of injectors, producers, and control measurements in the case study. As the control intervals increase and the time horizon extends, the number of constraints grows, making the optimization problem more complex.

For the Egg model, with 8 injectors, 4 producers, and a 0.5-year horizon with weekly adjustments (26 measurements), the 416 output constraints are derived as follows: eqs 8 and 9 define 312 flow rate constraints applied to each injector and producer at every time step. Equations 10–12 contribute 78 platform capacity constraints, ensuring that the total injection and production rates remain within platform limits. Finally, eq 13 accounts for 26 water-cut constraints, one for each time step.

In comparison, the UNISIM case study, with 11 injectors, 14 producers, and a 5-year horizon with monthly adjustments (61 measurements), results in 1,769 output constraints. This includes 1,525 flow rate constraints derived from eqs 8 and 9, 183 platform capacity constraints from eqs 10–12, and 61 water-cut constraints from eq 13. This detailed breakdown demonstrates how the number of constraints scales with the number of injectors, producers, and control measurements, increasing the complexity of the optimization problem.

The detailed list of parameters used in this analysis, along with a comparison of the problem’s dimensionality with and without the FCM approach, is provided in Table S1 for the Egg model and Table S2 for the UNISIM model in the Supporting Information.

### 2.3 Computational Framework

**Figure 3:**
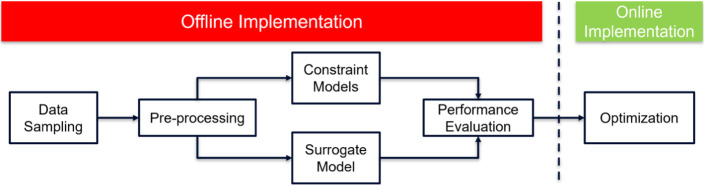
Computational framework.

This section outlines the computational framework employed in our study, as illustrated in Figure 3. The overall framework consists of both offline and online implementations.

In the offline phase, we collect and preprocess data, which is crucial for building an accurate surrogate reservoir model. This model is then tuned to ensure reliability and performance. Additionally, classification models are developed to handle constraints effectively, and both surrogate and classification models are saved for use in the online phase without further retraining.

In the online implementation, the preprocessed data and developed models are utilized to optimize the problem using data-driven optimizers. This approach ensures that the optimization process is both efficient and effective, leveraging the strengths of surrogate modeling and classification for constraint management.

The offline implementation focuses on preparing and refining models, while the online implementation applies these models for real-time optimization. Each of these phases will be explained in detail in the subsequent subheadings. This overall framework ensures a comprehensive approach to optimizing waterflooding operations, balancing computational efficiency with the accuracy and feasibility of the solutions generated.

#### 2.3.1 Offline Implementation

##### 2.3.1.1 Data Sampling

We begin with comprehensive data collection to model and optimize the waterflooding process. Utilizing a maximin latin hypercube design (LHD),^67^ we generate 20,000 parametrized BHP samples with the PyDOE library in Python. This sampling technique ensures a well-distributed data set that thoroughly covers the input space, reducing gaps and improving model accuracy. The maximin criterion maximizes the minimum distance between sample points, making it particularly effective for high-dimensional problems, such as the 75 input variables in the UNISIM model and 36 in the Egg model. This efficient and structured approach allows for thorough exploration of the parameter space, ensuring robustness and generalizability in our models.

These parametrized BHP samples are then processed through the MATLAB Reservoir Simulation Toolbox (MRST), an open-source tool developed by SINTEF for reservoir simulation.^68,69^ MRST solves partial differential equations governing multiphase flow in porous media. For immiscible fluids, the conservation equation is expressed as
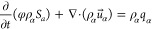
15

where φ is porosity, ρ_α_ is fluid density, *S*_a_ is saturation, *u⃗*_α_ is Darcy velocity, and *q*_α_ represents sources or sinks. The extended Darcy’s law for multiphase flow is given by^60^
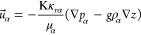
16

Here, *K* is absolute permeability, *κ*_ra_ is relative permeability, μ_α_ is fluid viscosity, ∇*p*_α_ is pressure gradient, and *g* is gravitational acceleration.

The MRST simulations output liquid flow rates for each BHP configuration. These flow rates are critical for evaluating the constraints governing the system’s operational feasibility. Using the flow rate values, we calculate the constraints as defined by eqs 8–13, covering flow rate limits, platform capacity, and water-cut conditions. Once these constraints are computed, each BHP configuration is assessed for feasibility: if all constraints are satisfied, the configuration is labeled as feasible; otherwise, it is labeled as infeasible. This labeling process ensures that feasibility is directly tied to the constraint evaluations derived from simulation results. After determining feasibility, the next step involves calculating the NPV as defined in eq 1. The NPV accounts for revenues from produced oil and the associated costs of water injection and handling, providing a comprehensive measure of economic performance across the entire production timeline. The NPV serves as the optimization objective, guiding the identification of optimal BHP settings for each well at different time intervals.

This comprehensive approach, combining advanced sampling techniques with high-fidelity reservoir simulation, enables precise modeling and optimization of waterflooding processes. By applying this methodology to different reservoir scenarios, we can evaluate its effectiveness and robustness across varying geological and operational conditions.

##### 2.3.1.2 Reservoir Surrogate Model

Surrogate models are critical in optimization problems to approximate the complex relationships between input and output variables.^70−72^ The choice of the surrogate model is crucial and depends on the specific problem and its complexity. Previously, other regression models were tested within the context of surrogate-based optimization for this problem.^45^ However, the problem was found to exhibit highly nonlinear and nonconvex behavior, making advanced techniques like neural networks necessary for accurate approximations.

**Figure 4:**
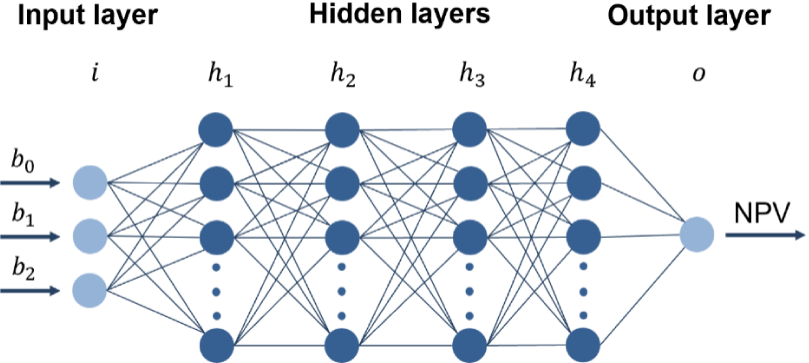
FFNN surrogate model architecture. Input layer: polynomial coefficients (b_0_,b_1_,b_2_) for well BHP profile. Output: single neuron for NPV. Hidden layer size and the number of neurons is determined after the hyperparameter tuning stage for each case study.

Given this complexity, we utilize a Feedforward Neural Network (FFNN) due to its proven ability to model nonlinear relationships effectively. An ANN, inspired by the human nervous system, excels at capturing empirical, nonlinear patterns within data. It consists of an input layer, one or more hidden layers, and an output layer (Figure 4). The number of hidden layers and neurons per layer is pivotal for balancing model capacity and preventing overfitting, where the model may fail to generalize well to unseen data.

The universal approximation theorem further justifies the use of ANNs, asserting that a neural network with at least one hidden layer can approximate any continuous function to any desired accuracy, provided it has enough neurons.^73^ This makes ANNs highly suitable for surrogate modeling. By leveraging their ability to model complex, nonlinear relationships, ANNs can provide accurate approximations that are essential for effective optimization and decision-making.

In this study, the surrogate model aims to predict the NPV over the production period based on the polynomial coefficients (*b*_0_,*b*_1_,*b*_2_) of for each well. The neuron size in the input layer changes depending on the number of injectors and producers. The output layer consists of a single neuron representing the NPV over the production period. We split the data set into training and testing sets in a 3:1 ratio. This split ensures that 75% of the data is available for training, providing sufficient information for the model to learn, while 25% is reserved for testing to robustly evaluate generalization performance. During training, we normalize the NPV data by scaling it to have a mean of zero and a standard deviation of one. This normalization is reversed after predictions to bring the values back to their original scale.

To fine-tune the model, we employ a 5-fold cross-validation strategy in which the data set is divided into five groups. In each iteration, one group serves as the validation set while the remaining groups form the training set, ensuring a comprehensive evaluation of the model’s performance and reducing the risk of overfitting. We specifically choose 5-fold cross-validation because it balances computational efficiency with robust performance estimation–while alternatives like 10-fold cross-validation or leave-one-out cross-validation are more thorough, they are also more computationally demanding. The final hyperparameters are chosen based on the configuration that achieves the best average performance across all five folds, thereby providing reliable and consistent results.

Using the training and validation data obtained from cross-validation, we construct a deep Feed-Forward Neural Network (FFNN) designed to map input BHP parameters to output flow data. The final network architecture – encompassing the number of hidden layers, the number of neurons per layer, and related hyperparameters such as batch size and number of epochs – is determined through an exhaustive grid search. Given the nonlinear nature of reservoir simulations, this architecture typically involves multiple hidden layers and a substantial number of nodes.

Hyperparameter optimization is critical to avoiding both underfitting and overfitting. For example, adjusting batch size influences how many training samples are processed before the model’s weights are updated, while tuning the number of epochs determines how many times the entire training set is passed through the network. To enhance the efficiency of training, we utilize the Adam optimizer, and we assess model performance using statistical indicators such as root mean square error (RMSE), mean square error (MSE), mean absolute error (MAE), and the coefficient of determination (*R*^2^).

After identifying the optimal hyperparameters through cross-validation, we rebuilt the surrogate model using the entire offline data set. This step allowed the model to compute its coefficients (its weights and biases) by fully leveraging all available data, thereby enhancing its robustness and accuracy for subsequent predictions.

Once the model is fine-tuned and achieves optimal performance by minimizing the MSE (the final hyperparameters of the neural network surrogate model are provided in Table S3), the neural network can quickly predict liquid flow rates for given BHP parameters. This results in immediate output retrieval in the online phase, significantly enhancing the efficiency and effectiveness of reservoir management by avoiding solving the complex differential equations at every iteration of data-driven optimization.

##### 2.3.1.3 Modeling the Feasible Region of the Reservoir Operation

In this task, we address constraint violations using a nonlinear classification algorithm. The total number of constraints in the optimization formulation depends on the number of discrete time points used to control the system. As the frequency of control and the length of the time horizon increase, so does the number of constraints. We propose that using accurate classifiers to model numerous process constraints at once and filter out infeasible solutions from the search space will enable the optimization algorithm to converge to feasible solutions in a computationally efficient manner consistently. This approach is anticipated to be more accurate than simple penalty functions or explicit constraint handling schemes. It does not require a feasible starting point as progressive barrier methods do, nor does it necessitate the modeling of each constraint with regression models.

**Figure 5:**
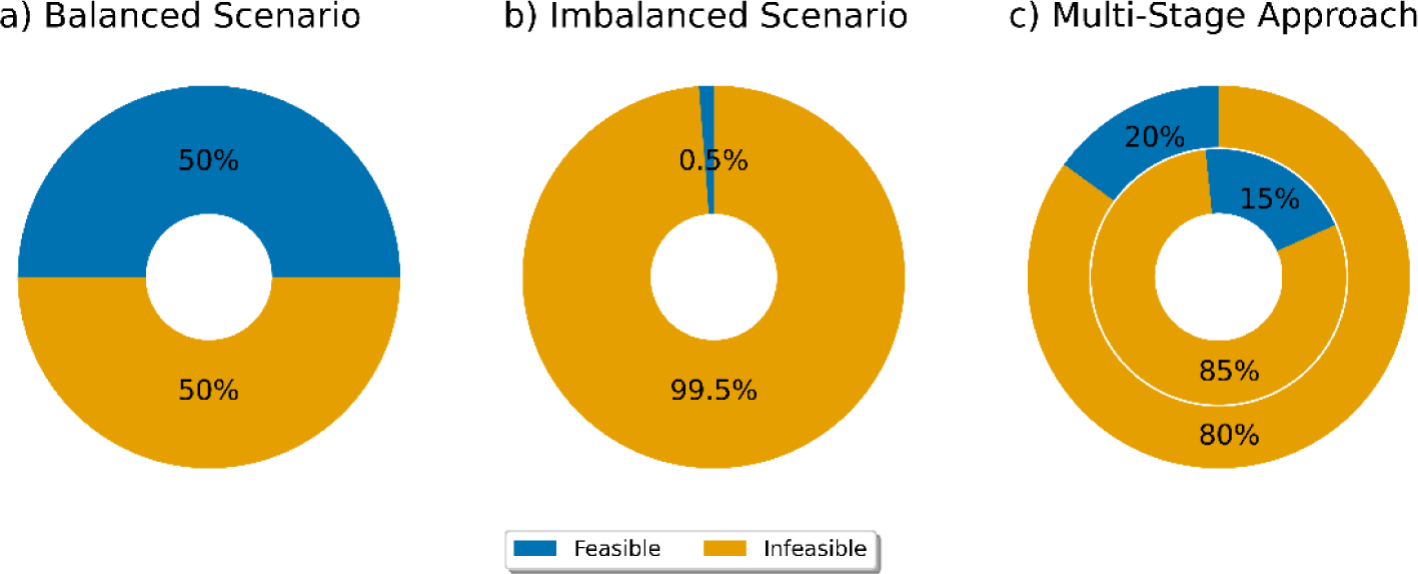
Examples of possible scenarios for class distribution in constraint classification – (a) Balanced case with feasible (blue) and infeasible (orange) points; (b) imbalanced case with underrepresented feasible points; (c) multistage classification using inner and outer doughnuts for two-stage feasibility assessment.

Each BHP setting is labeled as feasible or infeasible based on the constraint evaluations discussed above. However, in practice, some constraints are inherently harder to satisfy, and it is rare to meet all constraints simultaneously (or vice versa). This challenge often results in imbalanced data sets, where the feasible class is severely underrepresented. To illustrate this, Figure 5 presents these possible scenarios:

(a) A perfectly balanced data set with 50–50 class distributions, which is ideal but rarely achievable in practice.
(b) A highly imbalanced case, where feasible points constitute only 0.5% of the data set, demonstrating the challenges posed by real-world scenarios.
(c) A multistage classification approach, where constraints are divided into groups and modeled sequentially.

The multistage classification approach addresses this imbalance by dividing constraints into manageable groups and developing separate classifiers for each group. For example, inner and outer doughnuts can represent different constraint sets. To satisfy all constraints, input data must successfully pass through multiple classifiers in sequence, with each stage verifying a specific subset of feasibility criteria. Only configurations that pass every stage are considered fully feasible. This approach efficiently eliminates infeasible solutions while maintaining a systematic framework for evaluating and satisfying complex constraints.

Additionally, we employ an adaptive train-test split strategy to address the imbalance during model training. In this strategy, the class with fewer samples contributes 80% of its data for training, with an equivalent number of samples selected from the majority class to maintain balance. The remaining data is reserved for testing. This method ensures a perfectly balanced training set, enabling robust model training, while the testing set retains the original class imbalance to provide realistic performance evaluations.

The data is then used to train a two-class classification model using 5-fold cross-validation. The performance of several classifiers, including *C*-parametrized support vector machine (SVM) with a Gaussian radial basis function kernel,^74,75^ FFNN, random forest (RF),^76^ and gradient boosting classifier (GBC),^77^ will be evaluated. RF and GBC classifiers provide feature importance scores based on the mean decrease in impurity (MDI), commonly referred to as Gini Importance, which reflects how much each feature reduces uncertainty (impurity) during classification.

To refine the models, we applied a feature selection process using a pretrained model initialized with the computed feature importance scores. Features with importance values meeting a specified threshold were retained, ensuring that only the most significant predictors were included. This led to the development of “modified RF” and “modified GBC” models, focusing on the most informative features identified by the original classifiers. Thus, we model the constraints using six different algorithms.

Hyperparameters for these models are optimized through an exhaustive grid search (the final hyperparameters of all classification algorithms are provided in Tables S4–S9). The predictive performance is evaluated using confusion matrices and precision–recall curve analysis, as accuracy alone can be misleading in cases of extreme class imbalance. The classifier with the highest area under the precision–recall curve is selected and saved for use in the online phase. This classifier will be capable of filtering any incoming samples as feasible or infeasible through a function execution, ensuring efficient and accurate constraint handling during optimization.

#### 2.3.2 Online Implementation

The objective of the online implementation phase is to optimize the waterflooding process by adjusting the polynomial coefficients of the parametrized BHP settings within their specified boundaries. This involves using both classification and regression models to handle constraints and evaluate the objective function.

For constrained optimization, the process is as follows:

1. Classifier Evaluation:
  - The classifier first evaluates the input *x* ϵ *X* ⊆ *R*^*n*^ to determine feasibility:
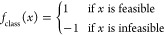
17

Here, *f*_class_:*X* → *Y* = {1,–1} is a function that divides the input space *X* into two classes.

2. Objective Function Assignment:
  - If the input is feasible (*f*_class_(*x*) = 1), the objective function *F*(*x*) is computed using the neural network surrogate model *f*_NN_(*x*)
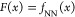
18
  - After obtaining the neural network prediction, we unscale it back to its original scale using our saved scaler.
  - If the input is infeasible (*f*_class_(*x*) = −1), the value of the objective function *F*(*x*) is automatically set to 0 as a penalty.

For bound-constrained optimization, the input data bypasses the classification step entirely and directly predicts the NPV using the neural network surrogate model. This streamlined approach skips the feasibility check since output constraints are not being evaluated, and the process is solely focused on optimizing the objective function within variable bounds. This iterative process continues until the convergence criteria are met, ensuring that the optimization algorithm refines the decision variables to achieve the optimal solution.

We employ both model-based and direct search-based optimizers, including ARGONAUT, NOMAD, Bayesian optimization, particle swarm optimization (PSO), and genetic algorithm.^78−86^ These optimizers iteratively adjust the variables, leveraging the strengths of surrogate modeling and classification to ensure an efficient and effective optimization process.^18^ By following this integrated approach, we enhance the efficiency and accuracy of the waterflooding optimization process, ensuring that the final solution is both feasible and optimized.

## 3 Results and Discussion

### 3.1 Predictive Performance of Reservoir Surrogate Model

Accurately predicting the NPV for various BHP settings is crucial in optimizing waterflooding operations. The performance of the reservoir surrogate model directly influences the efficiency and reliability of the optimization process, making it a pivotal component of this study.

We utilized a deep FFNN as the surrogate model to map the input BHP parameters to the output NPV values. The model was trained on a data set generated using the maximin LHD, ensuring a diverse and comprehensive representation of possible scenarios. The data set was split into training and testing sets in a 3:1 ratio to accurately evaluate the reservoir surrogate model’s performance. During training, we employed 5-fold cross-validation to tune the hyperparameters based on the MSE loss function, ensuring the model’s robustness and preventing overfitting.

**Figure 6:**
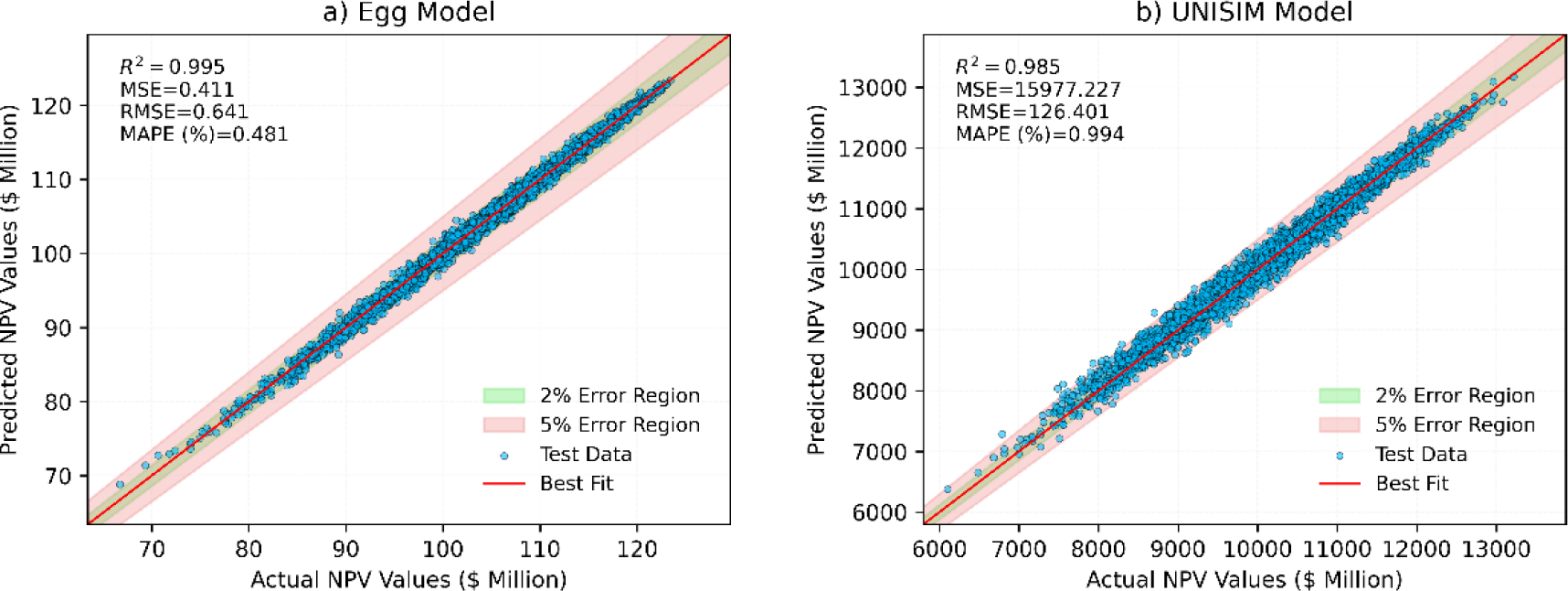
Predictive capability of the (a) Egg and (b) UNISIM reservoir surrogate models. Parity plots showing predicted vs actual NPV values for the test sets, with 2% and 5% error regions and performance metrics.

The surrogate model’s predictive accuracy was assessed using several statistical indicators. The testing results, depicted in the parity plot for the test set (Egg model), demonstrate an *R*^2^ value of 0.995, an MSE of 0.411, an RMSE of 0.641, and a MAPE of 0.481% (Figure 6). These metrics indicate a high level of accuracy in the model’s predictions. Notably, the majority of the points lie within the 2% error region, as shown in the plot.

**Figure 7:**
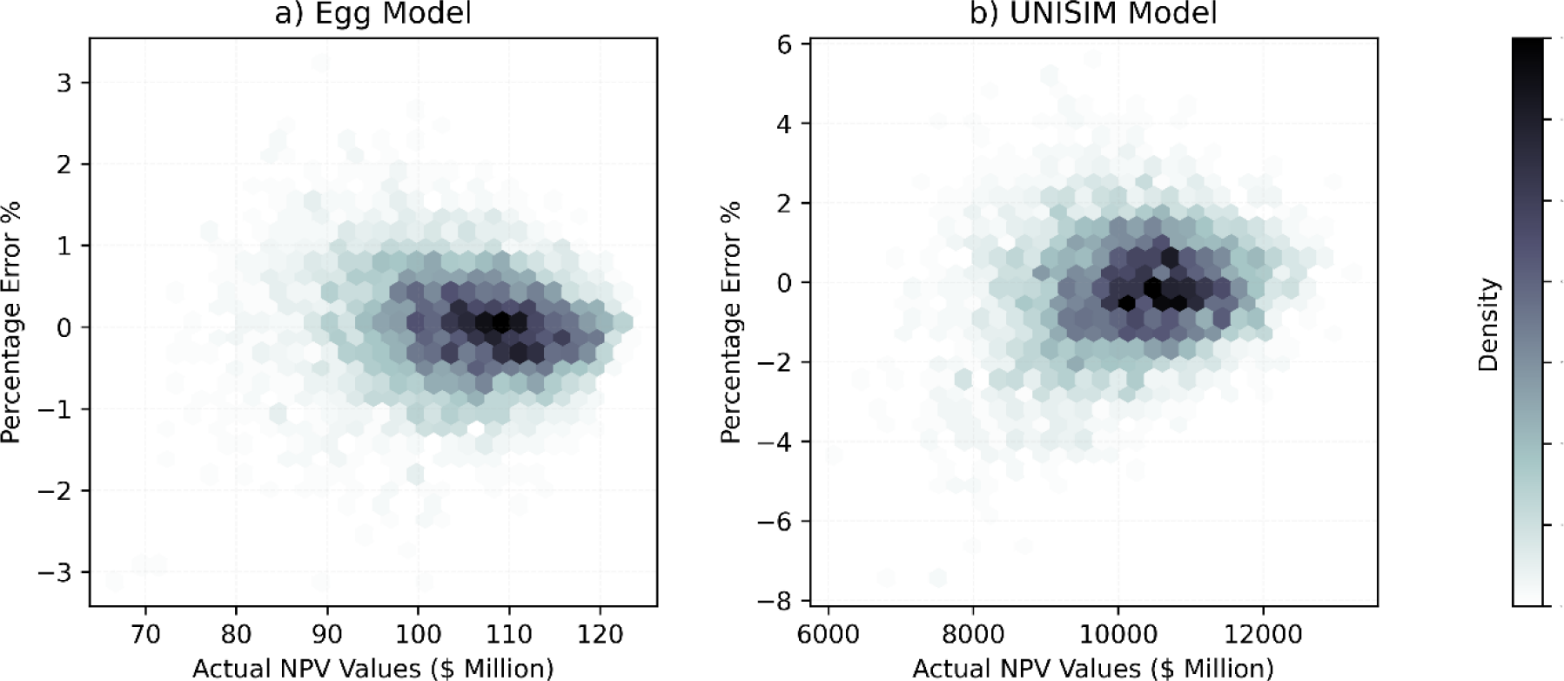
Hexbin plots of actual NPV values vs percentage error. The plots show the relationships between actual NPV values and percentage error for the test sets, with color intensity indicating data point density. Darker areas represent higher densities.

Furthermore, we achieved similarly significant results for a more complex case study – the UNISIM model. Despite its higher dimensionality, the surrogate model attained an *R*^2^ value of 0.985 and a MAPE of 0.994%. The hexbin plot in Figure 7 illustrates the actual NPV values against the percentage error for Egg model, further validating the model’s precision by showing the density of predictions. The color intensity represents the density of data points, with darker regions indicating higher densities. Most predictions fall within a narrow error band, with most points clustered around the 0% error line, confirming the model’s robustness and reliability. This plot highlights the model’s capability to deliver accurate predictions with minimal deviation consistently.

In addition to the strong performance observed on the test set, we further refined our surrogate model by retraining it on the complete data set. After determining the optimal hyperparameters through cross-validation, we re-estimated the final model coefficients using the entire data set. This final retraining allowed the model to fully exploit all available data, resulting in a more robust and precise surrogate model for online optimization.

Overall, the reservoir surrogate model exhibits exceptional performance in predicting NPV values, enabling efficient and accurate optimization of waterflooding operations. This accuracy ensures the optimization algorithm can reliably navigate the complex search space, identifying the optimal well-control settings to maximize economic returns.

### 3.2 Classification Performance of Reservoir’s Feasible Region

In reservoir management, handling constraints is crucial to ensure feasible operations and avoid impractical solutions. For our study, the collected data is labeled as feasible (1) or infeasible (−1) before being processed by six different classifiers. However, the data can be highly imbalanced due to the rarity of certain feasible conditions. This issue is exemplified by the UNISIM model, where feasible well settings are extremely scarce.

**Figure 8:**
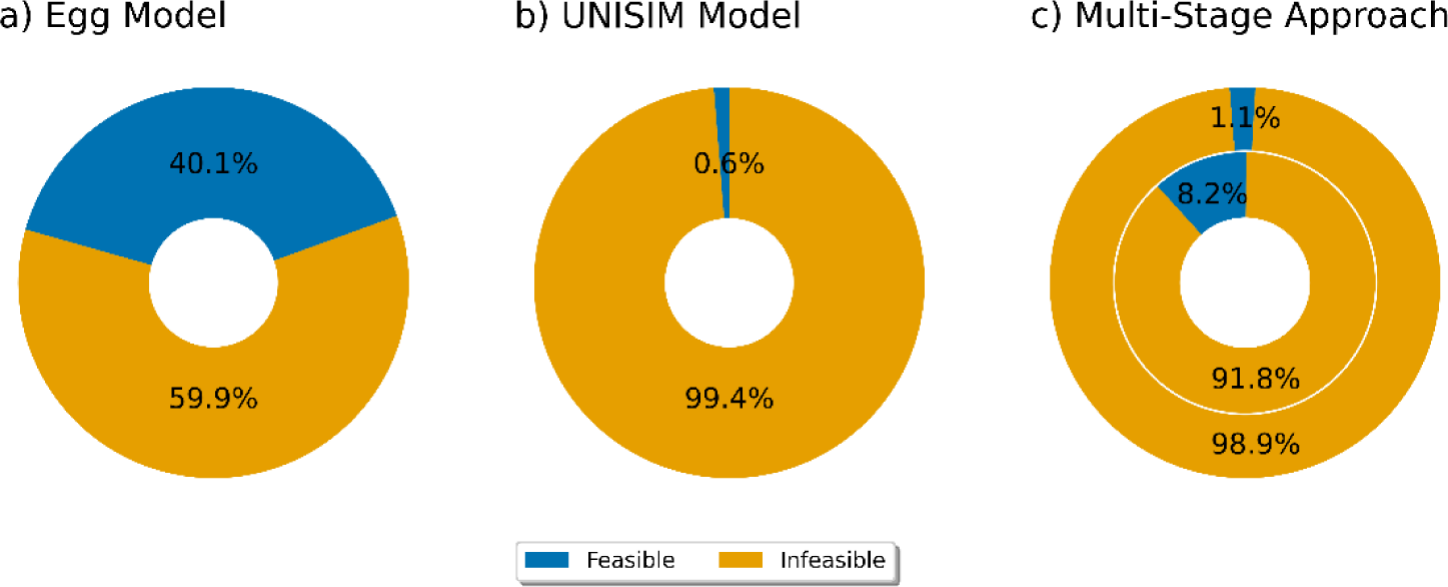
Class distribution and multistage classification approach – (a) Egg model with feasible (blue) and infeasible (orange) points; (b) UNISIM model with a significant class imbalance; (c) multistage classification for the UNISIM model showing inner and outer doughnuts for two-stage feasibility assessment to manage class imbalance.

Figure 8 illustrates the class distribution for constraint violations in both the Egg and UNISIM models, where blue indicates feasible points and orange indicates infeasible points. As shown in part (a), the Egg model has a more balanced distribution, with 40.1% feasible and 59.9% infeasible points. In contrast, part (b) highlights the significant class imbalance in the UNISIM model, where only 0.6% of the data points are feasible, making it challenging to identify the feasible regions.

As described previously (Section 2.3.1.3), we employed a multistage classification approach to address the issue of extreme class imbalance. In this approach, the first stage (inner doughnut) assesses feasibility based on the constraints in eqs 9–14. This classification step identifies 8.2% of the data points as feasible for the inner doughnut, as shown in part (c) of Figure 8. This initial stage captures the most critical constraints while increasing the representation of feasible points in the data set.

The second stage (outer doughnut) evaluates the data points labeled as feasible in the first stage against the water flow rate constraints for injectors (eq 8). This additional filtering step narrows down the feasible region further, ensuring that only the most restrictive feasible conditions are satisfied.

The overlap of these two stages results in the final feasible area. If a data point is classified as feasible by both stages, it belongs to 0.6% of the original feasible area. This multistage approach, visualized by the inner and outer doughnuts, allows us to manage the extreme class imbalance by ensuring more data points in both classes during classification. This step-by-step narrowing down helps accurately identify the feasible regions within the high-dimensional constraint space. We use an adaptive train-test split based on class imbalance when building these classifiers. Evaluating classifier performance in imbalanced data sets requires more than just accuracy, as accuracy can be misleading. For example, a classifier might achieve high accuracy by predicting the majority class most of the time, but this does not necessarily mean it performs well on the minority class. Therefore, we also evaluate sensitivity (recall), specificity, and balanced accuracy to provide a comprehensive assessment. In this context, true positives (TP) refer to instances where the positive class is correctly identified, while true negative (TN) refer to correctly identified instances of the negative class. False positives (FP) are negative instances that are incorrectly classified as positive, and false negatives (FN) are positive instances that are incorrectly classified as negative. These metrics are calculated as follows
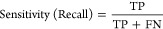
19
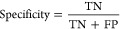
20
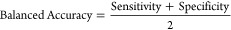
21

Sensitivity quantifies the model’s ability to correctly identify positive samples, while specificity measures its ability to correctly classify negative samples. Balanced accuracy provides a comprehensive view by averaging sensitivity and specificity, ensuring equal emphasis on both classes, which is particularly important for imbalanced data sets.The testing classification performances of various algorithms have been summarized in Table 1 extensively.

**Table 1:** Classification Metrics – Accuracy, Sensitivity, Specificity, Balanced Accuracy (in %) for Classification Models Used in Constraint Handling for the EGG and UNISIM Models across Various Algorithms^a^

		SVM	RF	GBC	RF2	GBC2	ANN
EGG	Accuracy	89.56	91.45	93.44	86.68	90.51	**93.74**
	Sensitivity	91.39	90.14	94.76	84.77	92.20	**96.00**
	Specificity	89.04	91.83	93.07	87.23	90.02	**93.08**
	Balanced Acc.	90.21	90.98	93.91	86.00	91.11	**94.54**
UNISIM (Inner)	Accuracy	84.54	83.70	**87.06**	83.64	83.94	83.15
	Sensitivity	93.94	**95.15**	93.03	92.12	92.12	81.82
	Specificity	84.36	83.48	**86.94**	83.48	83.78	83.17
	Balanced Acc.	89.15	89.32	**89.99**	87.80	87.95	82.50
UNISIM (Outer)	Accuracy	85.71	88.93	88.80	88.95	**90.00**	82.43
	Sensitivity	81.40	**97.67**	**97.67**	90.70	93.02	88.37
	Specificity	85.72	88.91	88.78	88.95	**89.99**	82.42
	Balanced Acc.	83.56	**93.29**	93.23	89.82	91.51	85.39

a The highest value for each metric within a scenario is highlighted in bold, indicating the best-performing algorithm for that specific category.

To get a clearer picture of performance in such imbalanced cases, we use precision and recall (sensitivity) metrics. Recall measures the proportion of actual positive instances that are correctly identified by the classifier, while precision quantifies the proportion of positive predictions that are correct. Precision is calculated as
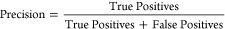
22

**Figure 9:**
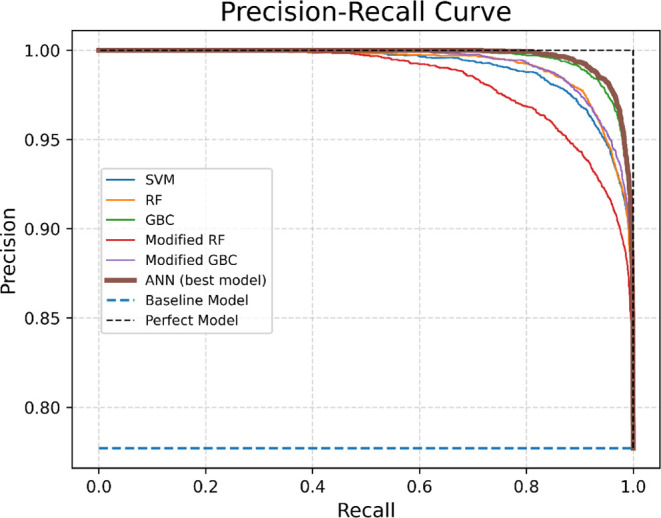
Precision–recall curve for classification algorithms (Egg model). This plot compares precision–recall performance of various algorithms for the Egg model. The neural network model, indicated by the thicker curve, is the best-performing.

Figure 9 illustrates the precision–recall curve (PR curve) for various classifiers in the context of constraint modeling for the EGG model, emphasizing the differences in their performance. The PR curve plots precision against recall across different probability thresholds. An ideal model would be represented by a point at (1,1), indicating perfect precision and recall. A skillful model is depicted by a curve that bows toward this point, reflecting high performance. In contrast, the baseline model, representing a no-skill classifier that predicts the majority class, is shown as a horizontal line, indicating random guessing.

The PR curve’s focus on the minority class makes it an effective diagnostic for imbalanced binary classification models. The precision–recall AUC (area under the curve) summarizes the curve with a single score, where 1.0 represents a model with perfect skill. The curve clearly shows that the neural network model outperforms other models, making it the best choice for the EGG case study. The baseline model, representing random guessing, shows significantly lower performance, underscoring the effectiveness of our approach. Precision–recall curves and confusion matrices for all classifiers are presented in Figures S1–S21.

When modeling the Egg reservoir constraints, the neural network outperformed other models due to the large training (12,816 data points) and testing (7,184 data points) data sets, which allowed it to fully leverage its capacity to learn complex, nonlinear patterns. However, for the UNISIM model, the significant class imbalance drastically reduced the effective training data size (2,636 data points for the inner stage and 338 data points for the outer stage, with the remaining data reserved for testing), leading to poorer performance from the neural network, as it is highly data hungry.

In contrast, decision tree-based models like GBC and RF performed better in the UNISIM model. GBC excelled in the inner stage, while RF was the best in the outer stage. These models are more robust with smaller, imbalanced data sets, due to their ensemble nature, which helps mitigate overfitting and effectively capture complex patterns. GBC consistently ranked in the top two across all applications, due to its ability to handle class imbalance. Meanwhile, SVM underperformed, possibly because the boundaries between feasible and infeasible regions were not well-defined or because the feasible region itself lay along complex boundaries, making it difficult for SVM to separate classes effectively.

### 3.3 Optimization Results

#### 3.3.1 Bound-Constrained Case

In the bound-constrained optimization scenario, the data points bypass the classification step and are directly processed by the regression model. The objective is to maximize the NPV by adjusting the polynomial coefficients of the BHP settings within their specified lower and upper bounds. The predicted NPV values are scaled back to their original units after being processed by the neural network surrogate model, and the optimization iteratively continues until convergence criteria are met.

We tested the performance of several optimization algorithms, including ARGONAUT, NOMAD, Bayesian optimization, particle swarm optimization (PSO), and genetic algorithm (GA), using the surrogate model for both the Egg and UNISIM cases. Each optimizer effectively refined the input variables, achieving convergence and maximizing the NPV. The surrogate model enabled rapid predictions, significantly reducing computational expense compared to traditional reservoir simulations, which may take several minutes per run depending on the case study. While there is a time overhead in building these surrogate models during the offline phase, the optimization process in the online phase becomes highly efficient. By leveraging the surrogate model, we can explore a larger solution space in a fraction of the time. Instead of running simulations that take minutes, the surrogate model allows us to predict outcomes within milliseconds during the online phase.

**Figure 10:**
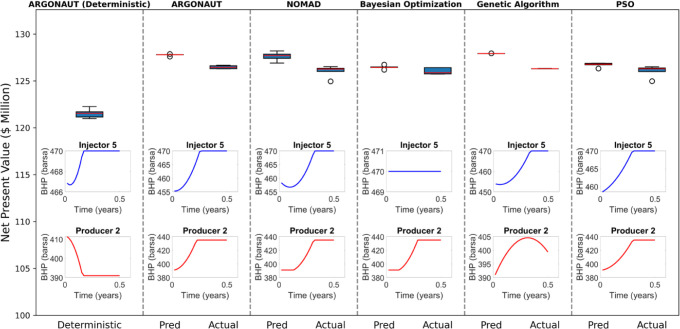
Optimal NPV and BHP profiles for various optimization algorithms (Egg model). This figure compares NPV distributions for different optimizers. The lower plots depict the control trajectories for the fifth injector and second producer, using polynomial coefficients of BHPs, over the production interval for the highest NPV case.

**Figure 11:**
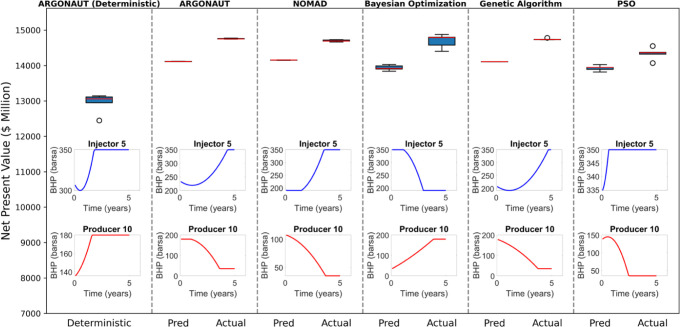
Optimal NPV and BHP profiles for various optimization algorithms (UNISIM model). This figure compares NPV distributions for different optimizers. The lower plots depict the control trajectories for the fifth injector and 10th producer, using polynomial coefficients of BHPs, over the production interval for the highest NPV case.

The results, as shown in Figure 10, indicate that for the bound-constrained case, the surrogate model not only accelerated the optimization process but also achieved higher NPV values. We optimized these processes with our computational framework using five different data-driven optimizers (ARGONAUT, Nomad, genetic algorithm, Bayesian optimization, and PSO). Also, we performed a deterministic optimization with the ARGONAUT framework, testing each approach over five runs for both the EGG and UNISIM models. ARGONAUT is an algorithmic framework designed for the global optimization of constrained grey-box problems. It incorporates techniques such as variable selection, bounds tightening, and constrained sampling to develop accurate surrogate representations of unknown equations, which are then globally optimized.^78−80^ To ensure a comprehensive comparison, we calculated each method’s average and maximum actual objective values from these five runs. The results for the EGG model, summarized in Figure 10, show that the ARGONAUT optimizer achieved the best performance, with up to a 4.06% improvement in average objective values and up to a 3.6% improvement in maximum values compared to the deterministic approach. For the UNISIM model, the results indicate that while ARGONAUT performed best on average, with improvements of up to 14.01%, the highest overall value was achieved by Bayesian Optimization with 13.21% improvement in comparison to the best objective value of the deterministic approach (Figure 11).

Although the results of the optimizers were generally similar, PSO performed slightly worse in the UNISIM case, with a 10.76% improvement in average values and a 10.72% improvement in maximum values compared to the deterministic method. The predicted NPV values were consistently very close to the actual NPV values, remaining within a 2% error region. All in all, our methodology for this bound-constrained case outperformed the deterministic approach, demonstrating its effectiveness in capturing the complex relationships within the reservoir data and optimizing the waterflooding process more efficiently.

#### 3.3.2 Constrained Case

In the constrained optimization scenario, output constraints of the reservoir simulation are managed using classifiers. While we achieve higher objective values in this case as well, the feasibility of specific BHP settings is highly dependent on the performance of the classifiers. Ideally, a perfect classifier would filter out infeasible BHP settings, but the presence of limited data and class imbalance poses significant challenges. This necessitates careful validation of the results to ensure reliability. In the Egg model, we received higher objective values than the deterministic case, but in some runs, infeasible objectives were observed. In the UNISIM model, having infeasible objectives was more common due to the more complex constraints and extreme class imbalance.

**Figure 12:**
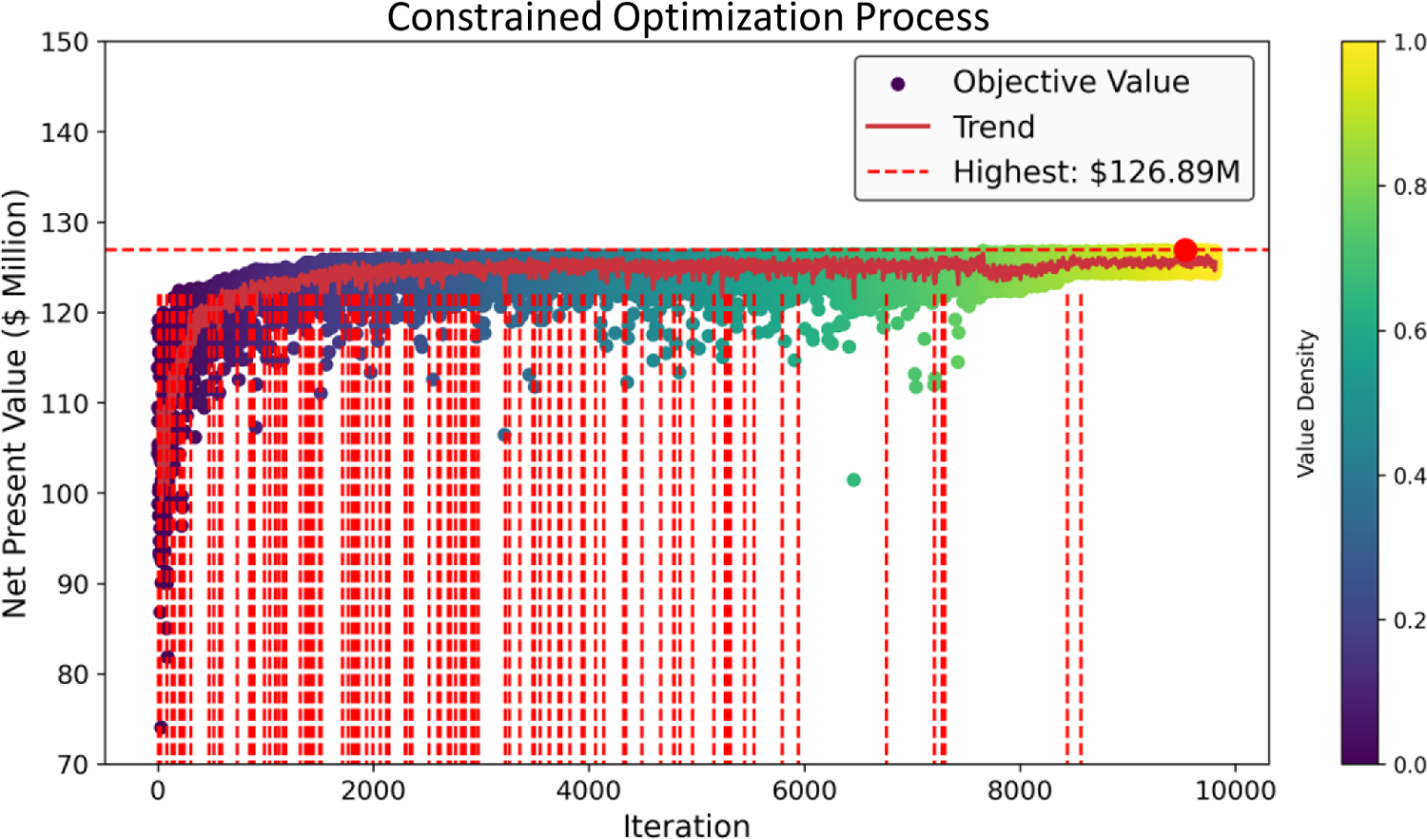
Constrained Optimization of Egg Model Using Genetic Algorithm. This plot shows the optimization process, with red vertical lines indicating infeasible input data points. The color bar shows value density, becoming brighter as values converge to a single optimal number.

Figure 12 demonstrates the optimization process using a genetic algorithm . The red vertical lines indicate infeasible input data points. As the generations progress, the infeasible samples are gradually eliminated, showing that the optimizer learns to search within feasible regions, ultimately converging to the optimal area.

We followed the same procedure for the UNISIM case, which is characterized by high dimensionality and complex constraints that are particularly challenging to handle. The extreme class imbalance in UNISIM necessitated a multistage classification approach. An input must pass through a double filter: it is first evaluated against the maximum water flow rate in m^3^/day, *q*_*w*_(*I*,*t*), that can be processed by each injector (eq 4), and if deemed feasible, it is then assessed against the remaining constraints. Only inputs classified as feasible by both stages are directed to the surrogate model. However, limited data and the necessity of passing through two classifiers, each with its own errors, reduce the accuracy of identifying feasible input data. This can lead to mistakenly determining feasible regions, which underscores the importance of validating the optimization results. Despite these challenges, the classifiers help manage constraints effectively, demonstrating the potential of our approach for complex reservoir management tasks. By integrating these classifiers within our optimization framework, we can significantly improve the efficiency of the waterflooding process, ensuring that the final solution is not only optimal but also feasible. While neural networks perform well with larger training sets, effectively capturing complex, nonlinear patterns, decision tree-based models like GBC and RF showed promising performance when the data was highly imbalanced and limited in size. This method showcases the importance of combining robust classification techniques with advanced optimization algorithms to handle high-dimensional, constrained optimization problems in reservoir management effectively.

## 4 Conclusions

This paper addresses the optimization of computationally expensive, high-dimensional, and highly constrained problems, focusing on the waterflooding process in secondary oil recovery using classification and regression algorithms. Such optimization is inherently challenging due to the complexity and high dimensionality of the problem space, coupled with the need to manage numerous constraints effectively. Our study presents a comprehensive methodology that integrates surrogate modeling and classification-based constraint handling, demonstrating its efficacy through two benchmark case studies: the relatively low-dimensional Egg model and the more complex, high-dimensional UNISIM model.

We reduce the problem’s high dimensionality by approximating BHP trajectories with second-order polynomial functions. By adjusting these polynomials, we derive liquid flow rates and calculate NPV, which accounts for revenue from produced oil and costs associated with water handling. The computational expense of traditional reservoir simulations is mitigated by constructing highly accurate surrogate models, achieving *R*^2^ values greater than 0.98 for both models. Constraints are managed by transforming the input space into feasible and infeasible regions using classification algorithms, allowing us to handle constraints simultaneously, regardless of their number.

Our methodology involves an offline phase for developing a regression model as a surrogate for reservoir simulations and a classification model for mapping the feasible region to handle constraints, followed by an online phase employing various data-driven optimizers to find optimal BHP settings. In bound-constrained scenarios, our approach outperformed the deterministic method, achieving higher NPV more efficiently. We tested the optimizers over 5 runs, and although the results of these optimizers were close to each other, ARGONAUT performed the best. Specifically, for the EGG model, ARGONAUT showed an improvement of up to 4.06% in average objective value and 3.6% in the maximum objective value across these 5 runs. Similarly, for the UNISIM model, ARGONAUT achieved improvements of up to 14.01% in the average objective value and 12.42% in the maximum objective value. This demonstrates the superior effectiveness of ARGONAUT in both case studies. For constrained cases, while we obtained higher objective values, solution feasibility depended heavily on the classifier performance. The UNISIM model highlighted challenges in scenarios with extreme class imbalance, necessitating a multistage classification approach and careful result validation. Classifiers were trained using an adaptive train-test split to ensure perfectly balanced training sets and evaluated using precision–recall curves. In the EGG model, the neural network excelled due to the larger data set, but in the more imbalanced and smaller UNISIM training data set, its performance declined. Instead, decision tree-based models like GBC and RF performed better, showing their effectiveness in handling limited and imbalanced data. However, in scenarios with rare feasible conditions, even with a highly accurate classifier, the objective function may converge on infeasible regions and violate some constraints. We observed this in the UNISIM case study, and in the Egg model, constraints were violated in a few iterations as well. This underscores the need for additional validation of optimization results, especially in highly constrained scenarios.

Through the use of surrogate models and classification-based constraint handling, our methodology minimizes computational demands while guaranteeing solutions that are both feasible and optimized. This innovative strategy enhances waterflooding optimization efficiency and has broader applications in managing highly constrained, computationally intensive processes. Our work demonstrates the potential of data-driven optimization to address complex real-world challenges, leading to more efficient engineering solutions.

## Supporting Information Available

Parameters and values used in both case studies (Egg and UNISIM models), hyperparameters for the trained classification and regression models, confusion matrices, precision–recall curves for all six algorithms across three different cases (PDF)
