# Supplementary material for: Surrogate-Assisted Optimization of Highly Constrained Oil Recovery Processes Using Classification-Based Constraint Modeling
Source: Ind Eng Chem Res. 2025 Apr 7;64(15):7751–66. doi: 10.1021/acs.iecr.4c03294 (PMC12007002; doi:10.1021/acs.iecr.4c03294)
Supplement: Supplementary file 1 — ie4c03294_si_001.pdf [file ie4c03294_si_001.pdf]

Supporting Information for “Surrogate-Assisted Optimization of Highly Constrained Oil Recovery Processes Using Classification-Based Constraint Modeling”

Zahir Aghayev[1,2], Dimitrios Voulanas[3,4], Eduardo Gildin[3], Burcu Beykal[1,2*]

[1]Department of Chemical and Biomolecular Engineering, University of Connecticut, Storrs, CT

[2]Center for Clean Energy Engineering, University of Connecticut, Storrs, CT

[3]Harold Vance Department of Petroleum Engineering, Texas A&M University, College Station, TX

[4] Texas A&M Energy Institute, Texas A&M University, College Station, TX

Table S1. Values of the parameters used in the Egg reservoir simulation and the dimensionality of the problem.

Parameters	*Value*
Oil price	50 $\$/stb$
$C_{I,W}$, $C_{P,W}$	1 $\$/stb$
d	0.09
n_T	26
n_I	8
n_P	4
BHP_I^{min}	450 $barsa$
BHP_I^{max}	470 $barsa$
BHP_P^{min}	391 $barsa$
BHP_P^{max}	435 $barsa$
q_W^{max}	1,100 m^3/day
q_{liq}^{max}	750 m^3/day
$Q_{I,W}^{max}$	3,750 m^3/day
$Q_{P,W}^{max}$	2,500 m^3/day
$Q_{P,O}^{max}$	2,500 m^3/day
Production horizon	0.5 year
Adjustment frequency	Weekly
Original control at the simulation level	312
FCM with 2nd order polynomial approximation	75
Number of total output constraints	416

Table S2. Values of the parameters used in the UNISIM reservoir simulation and the dimensionality of the problem.

Parameters	*Value*
Oil price	50 $\$/stb$
$C_{I,W}$, $C_{P,W}$	1 $\$/stb$
d	0.09
n_T	61
n_I	11
n_P	14
BHP_I^{min}	190 $barsa$
BHP_I^{max}	350 $barsa$
BHP_P^{min}	35 $barsa$
BHP_P^{max}	180 $barsa$
q_W^{max}	9,000 m^3/day
q_{liq}^{max}	1,500 m^3/day
$Q_{I,W}^{max}$	38,156 m^3/day
$Q_{P,W}^{max}$	21,240 m^3/day
$Q_{P,O}^{max}$	21,240 m^3/day
Production horizon	5 years
Adjustment frequency	Monthly
Original control at the simulation level	1,525
FCM with 2nd order polynomial approximation	75
Number of total output constraints	1,769

Table S3. Final Hyperparameters for Neural Network Surrogates in Egg and UNISIM case studies.

Scenario	*Hidden layer 1*	*Hidden layer 2*	*Hidden layer 3*	*Hidden layer 4*	*Activation Function*	*Learning Rate*	*Batch Size*	*Epochs*
Egg	96	32	32	64	Sigmoid	0.0016	64	1000
UNISIM	64	64	96	96	Sigmoid	0.0016	64	1000

Table S4. Final Hyperparameters for SVM classifiers in Egg and UNISIM case studies.

Scenario	*Cost*	*Gamma*
Egg Model	4000	1/1500
UNISIM (Inner)	300	1/15000
UNISIM (Outer)	2500	1/15000

Table S5. Final Hyperparameters for RF classifiers in Egg and UNISIM case studies.

Scenario	*Max Depth*	*Min Samples Leaf*	*Min Samples Split*	*N estimators*
Egg Model	16	1	2	100
UNISIM (Inner)	12	4	2	500
UNISIM (Outer)	4	1	16	500

Table S6. Final Hyperparameters for GBT classifiers in Egg and UNISIM case studies.

Scenario	*Learning Rate*	*Max Depth*	*Min Samples Leaf*	*Min Samples Split*	*N estimators*
Egg Model	0.1	8	6	64	200
UNISIM (Inner)	0.1	2	8	64	500
UNISIM (Outer)	0.25	2	6	2	25

Table S7. Final Hyperparameters for Modified RF classifiers in Egg and UNISIM case studies.

Scenario	*Max Depth*	*Min Samples Leaf*	*Min Samples Split*	*N estimators*
Egg Model	8	8	32	75
UNISIM (Inner)	None	7	2	75
UNISIM (Outer)	None	4	2	5

Table S8. Final Hyperparameters for Modified GBT Classifiers in Egg and UNISIM case studies.

Scenario	*Learning Rate*	*Max Depth*	*Min Samples Leaf*	*Min Samples Split*	*N estimators*
Egg Model	0.1	4	7	64	100
UNISIM (Inner)	0.1	4	1	64	100
UNISIM (Outer)	0.05	4	8	2	200

Table S9. Final Hyperparameters for ANN classifiers in Egg and UNISIM case studies.

Scenario	*Hidden Layer 1*	*Hidden Layer 2*	*Hidden Layer 3*	*Hidden Layer 4*	*Hidden Act. Func.*	*Output Act. Func.*	*Learning Rate*	*Batch Size*	*Epochs*
Egg Model	12	16	32	16	tanh	sigmoid	0.0008	64	100
UNISIM (Inner)	12	32	16	32	tanh	sigmoid	0.0016	64	100
UNISIM (Outer)	32	12	12	64	tanh	sigmoid	0.0008	64	100

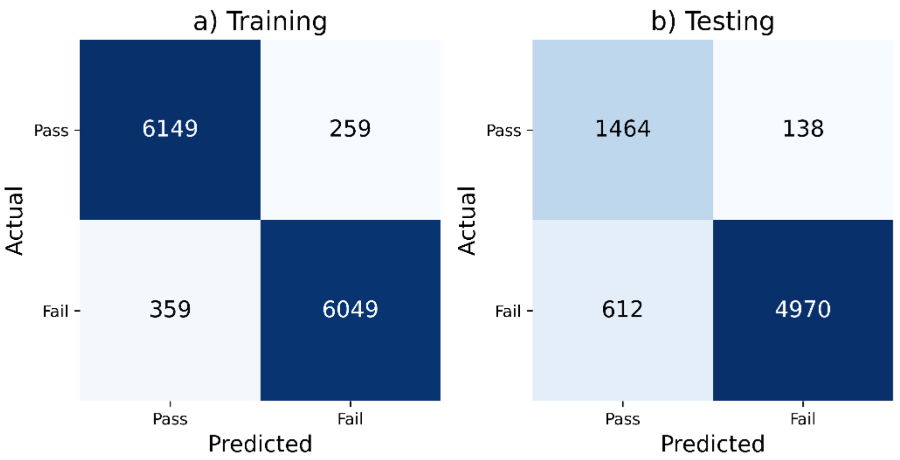

Figure S1. Confusion matrix of the SVM classifier for the Egg model. (a) Training set. (b) Testing set.

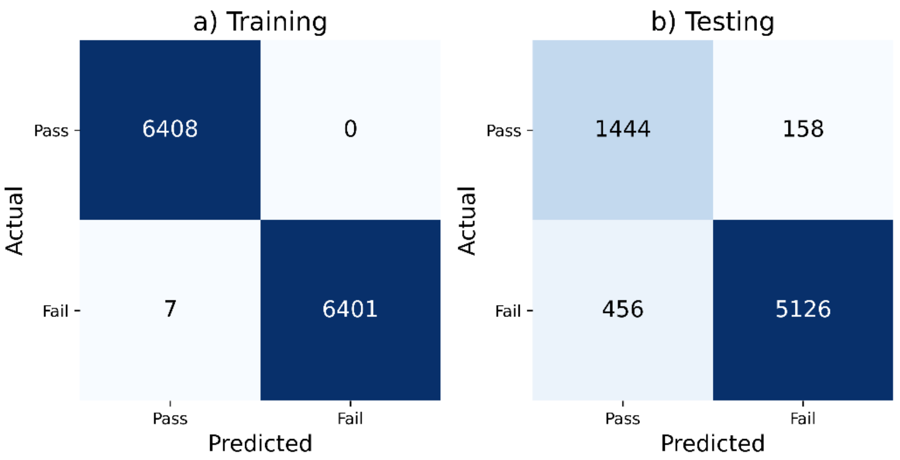

Figure S2. Confusion matrix of the RF classifier for the Egg model. (a) Training set. (b) Testing set.

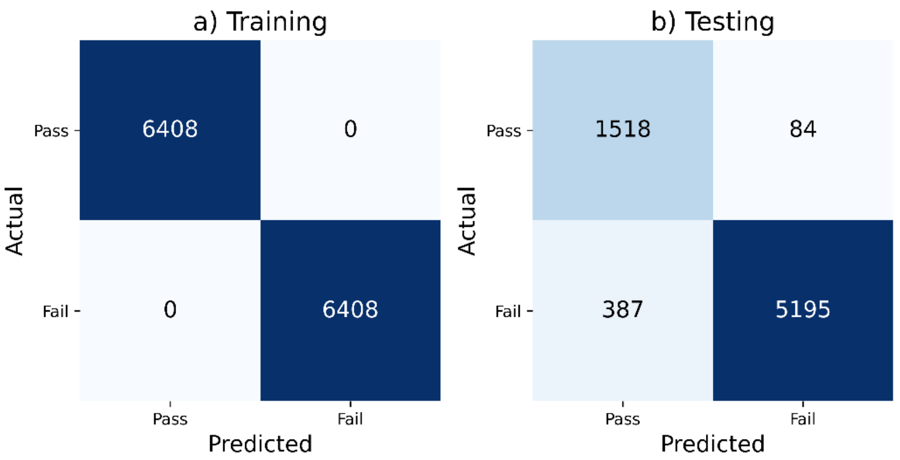

Figure S3. Confusion matrix of the GBT classifier for the Egg model. (a) Training set. (b) Testing set.

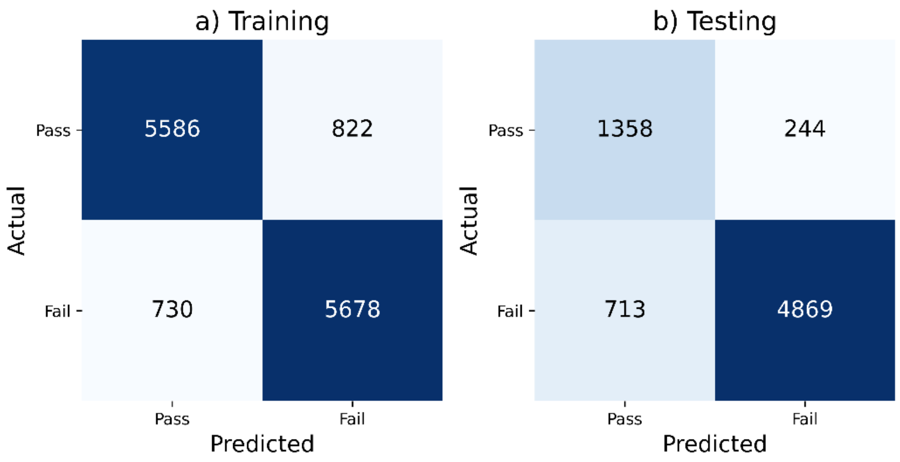

Figure S4. Confusion matrix of the modified RF classifier for the Egg model. (a) Training set. (b) Testing set.

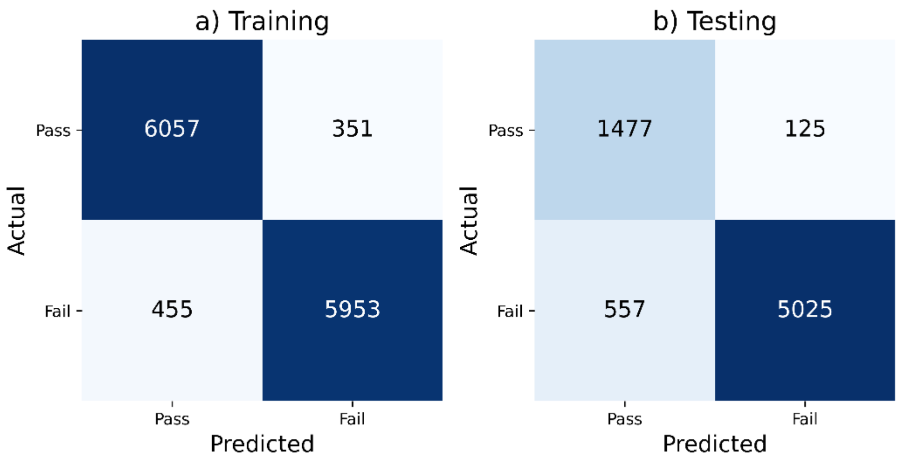

Figure S5. Confusion matrix of the modified GBT classifier for the Egg model. (a) Training set. (b) Testing set.

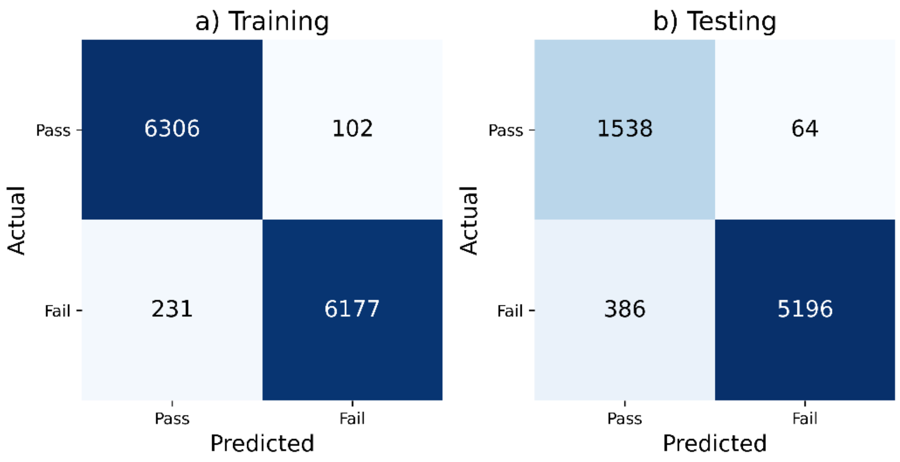

Figure S6. Confusion matrix of the ANN classifier for the Egg model. (a) Training set. (b) Testing set.

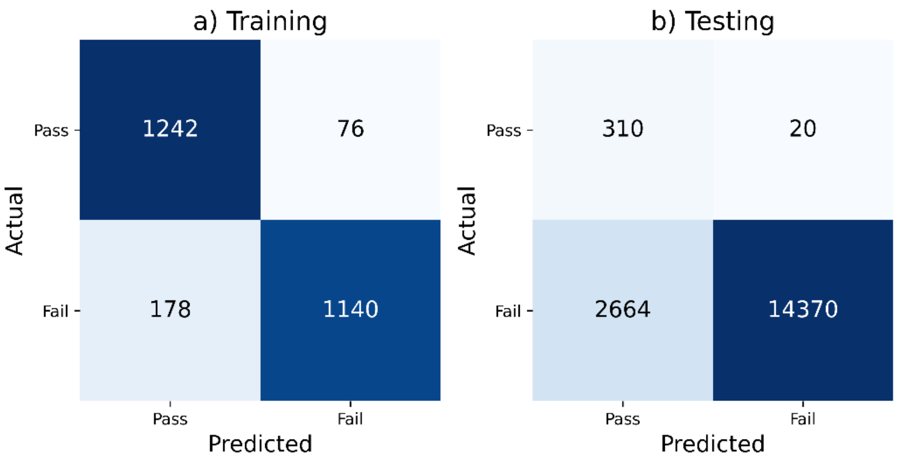

Figure S7. Confusion matrix of the SVM classifier for the inner stage of UNISIM model. (a) Training set. (b) Testing set.

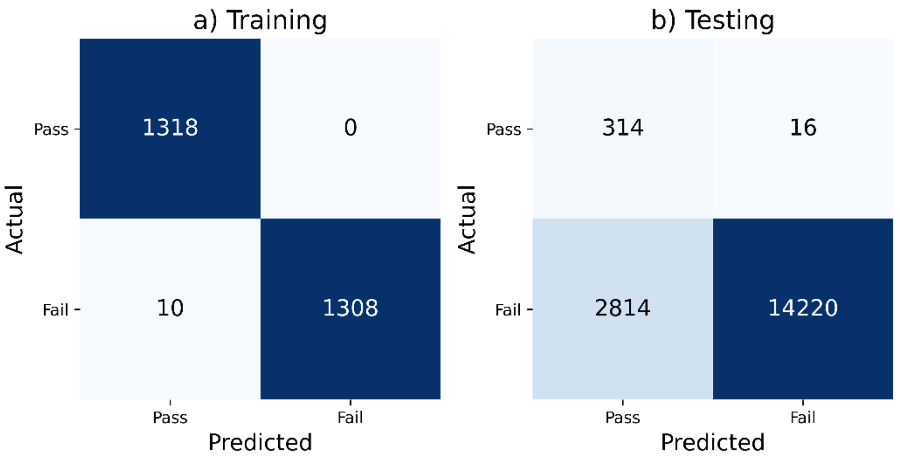

Figure S8. Confusion matrix of the RF classifier for the inner stage of UNISIM model. (a) Training set. (b) Testing set.

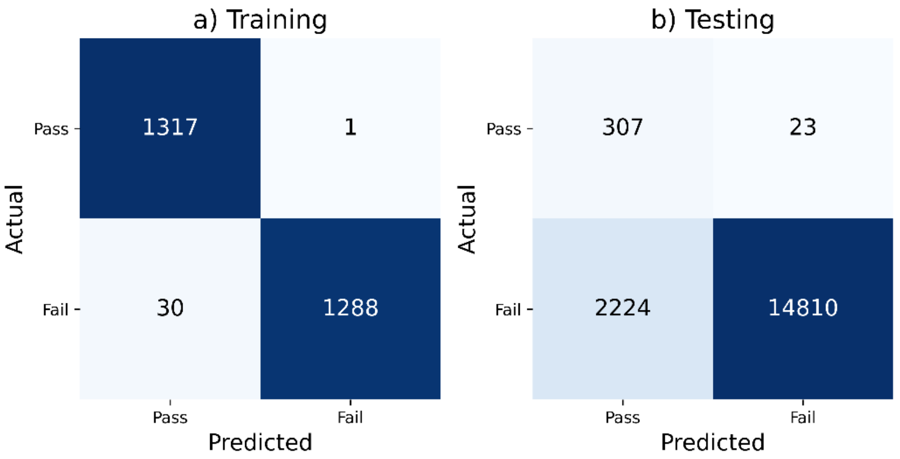

Figure S9. Confusion matrix of the GBT classifier for the inner stage of UNISIM model. (a) Training set. (b) Testing set.

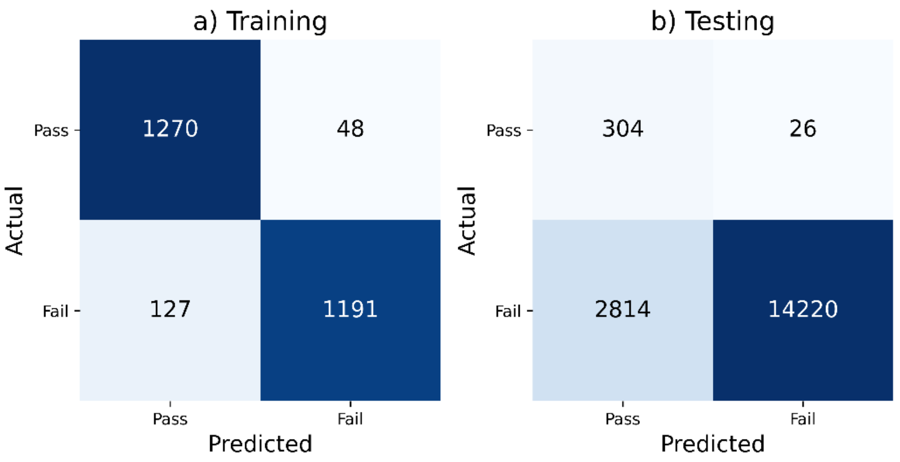

Figure S10. Confusion matrix of the modified RF classifier for the inner stage of UNISIM model. (a) Training set. (b) Testing set.

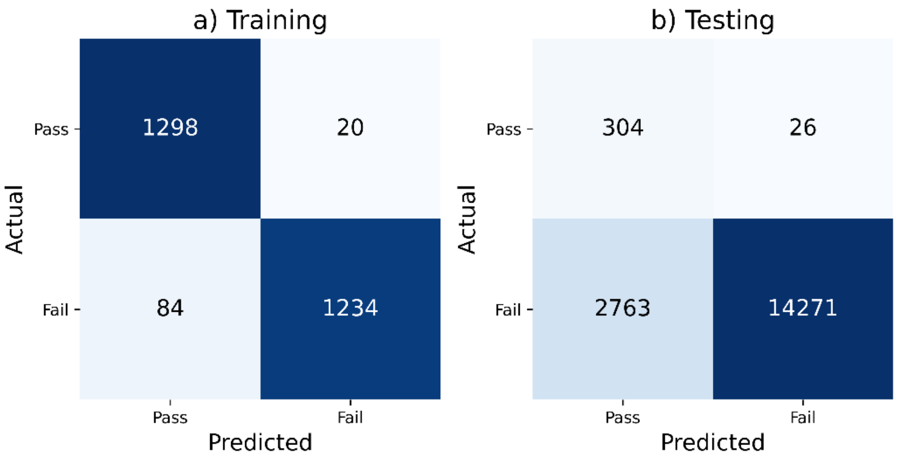

Figure S11. Confusion matrix of the modified GBT classifier for the inner stage of UNISIM model. (a) Training set. (b) Testing set.

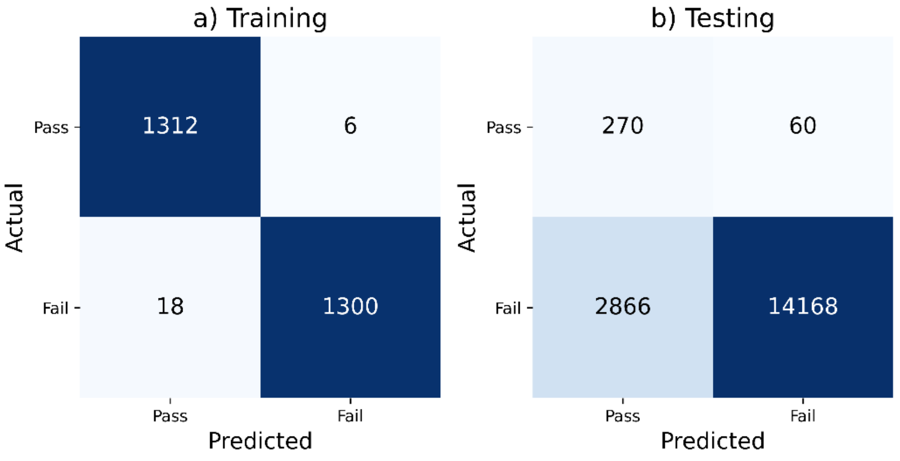

Figure S12. Confusion matrix of the ANN classifier for the inner stage of UNISIM model. (a) Training set. (b) Testing set.

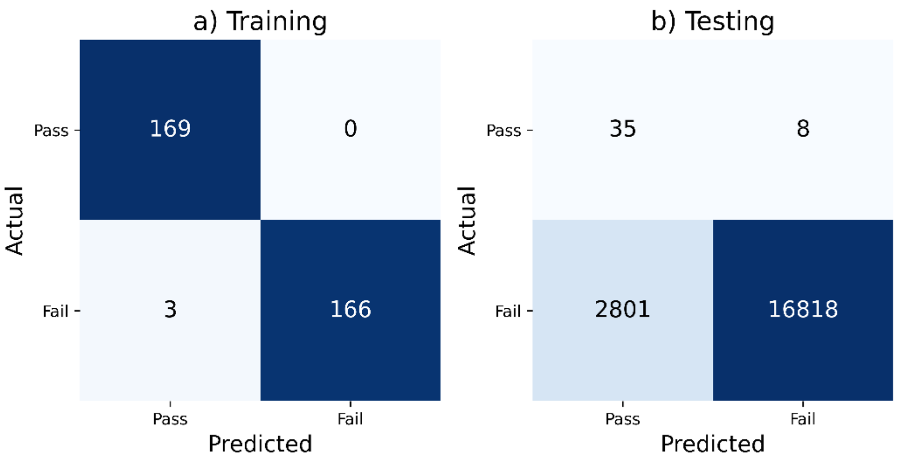

Figure S13. Confusion matrix of the SVM classifier for the outer stage of UNISIM model. (a) Training set. (b) Testing set.

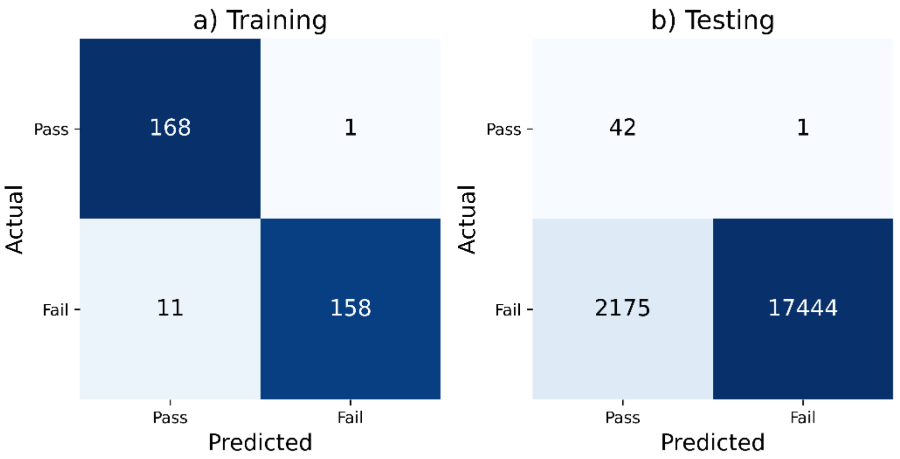

Figure S14. Confusion matrix of the RF classifier for the outer stage of UNISIM model. (a) Training set. (b) Testing set.

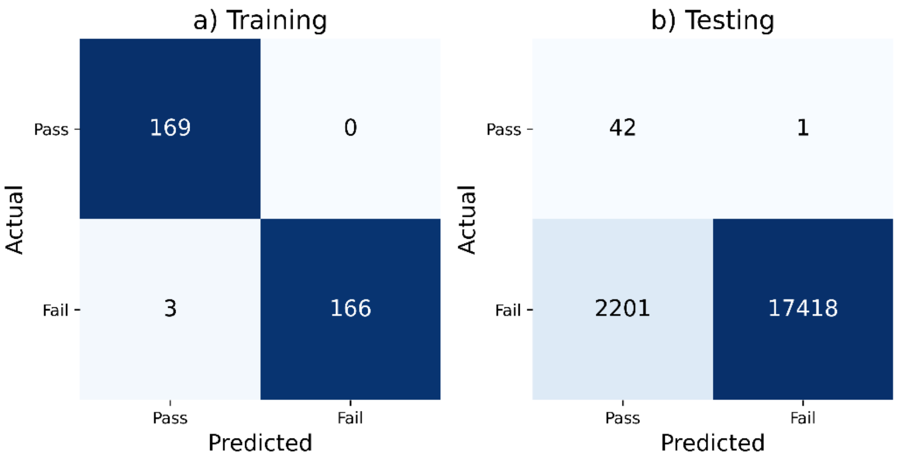

Figure S15. Confusion matrix of the GBT classifier for the outer stage of UNISIM model. (a) Training set. (b) Testing set.

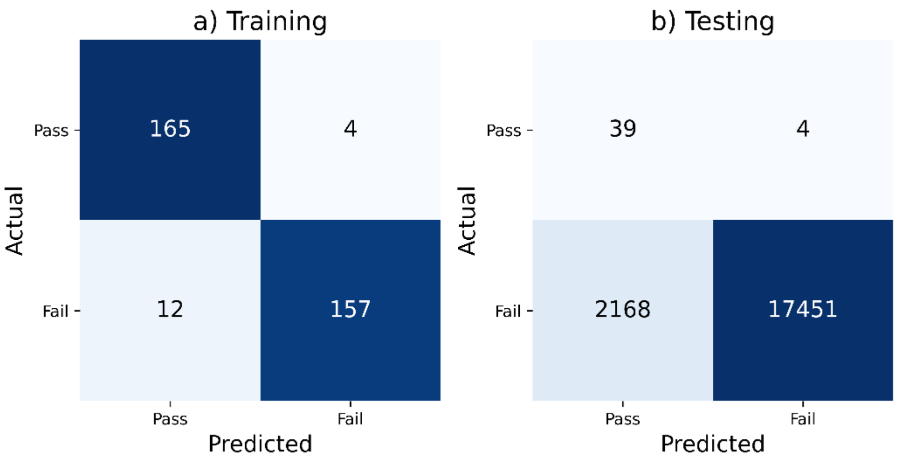

Figure S16. Confusion matrix of the modified RF classifier for the outer stage of UNISIM model. (a) Training set. (b) Testing set.

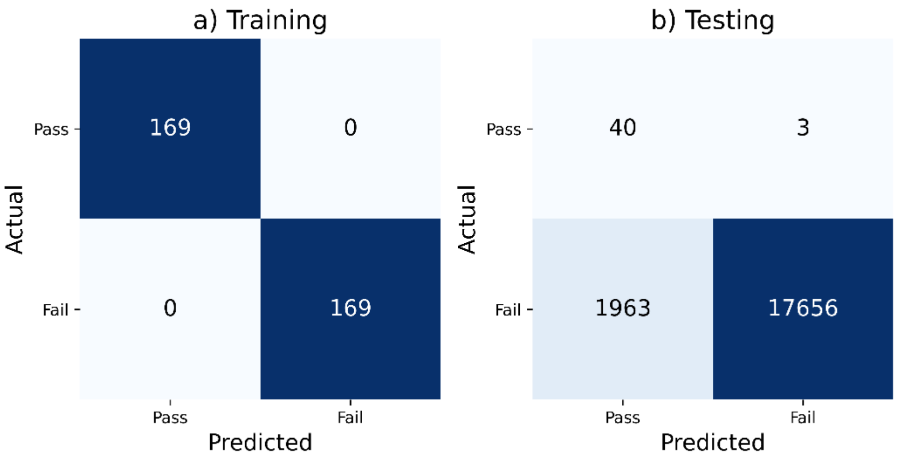

Figure S17. Confusion matrix of the modified GBT classifier for the outer stage of UNISIM model. (a) Training set. (b) Testing set.

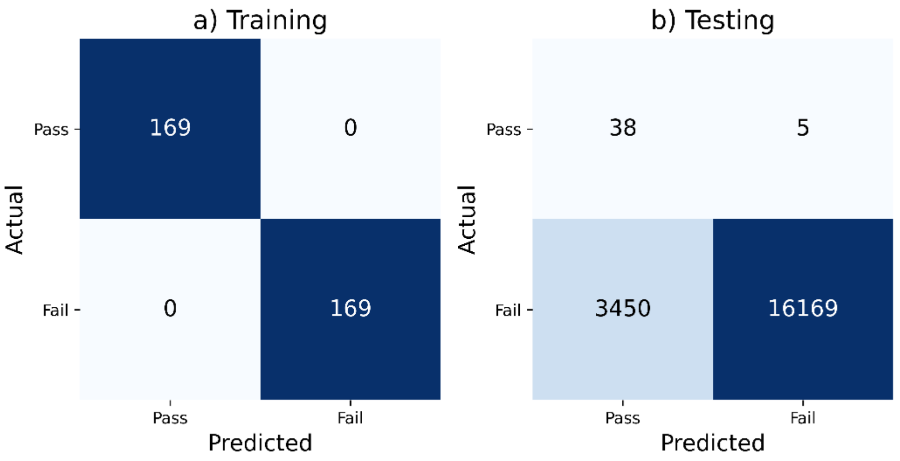

Figure S18. Confusion matrix of the ANN classifier for the outer stage of UNISIM model. (a) Training set. (b) Testing set.

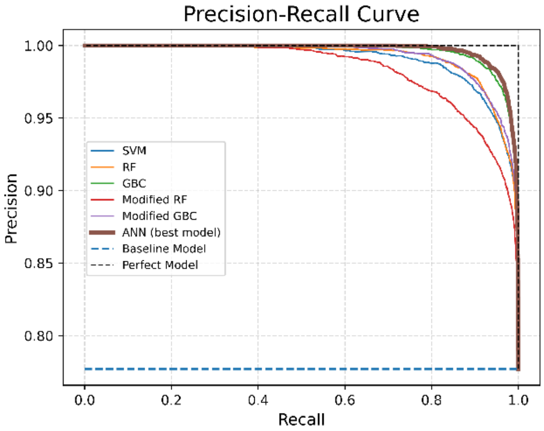

Figure S19. Precision-recall curve for classification algorithms (Egg model). The neural network model, indicated by the thicker curve, is the best-performing.

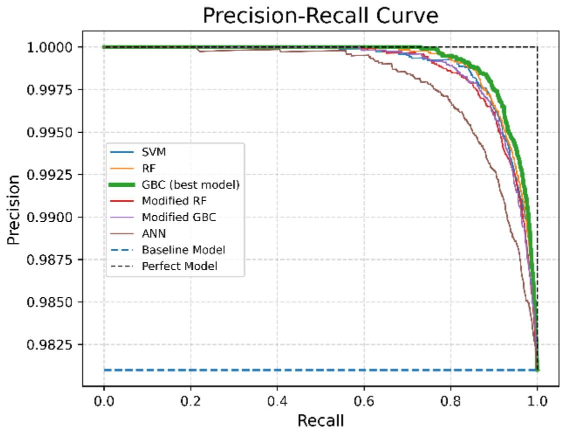

Figure S20. Precision-recall curve for classification algorithms (inner stage of UNISIM model). The gradient boosting classifier model, indicated by the thicker curve, is the best-performing.

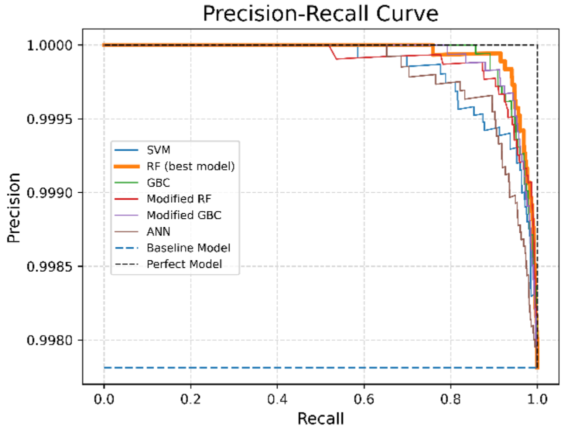

Figure S21. Precision-recall curve for classification algorithms (outer stage of UNISIM model). The random forest model, indicated by the thicker curve, is the best-performing.
